# A Glycoproteome Data Mining Strategy for Characterizing Structural Features of Altered Glycans with Thymic Involution

**DOI:** 10.1002/advs.202502013

**Published:** 2025-07-24

**Authors:** Zhida Zhang, Yongqi Wu, Ke Hou, Yiwen Zhang, Lin Chen, Muyao Yang, Zhehui Jin, Yongchao Xu, Yingjie Zhang, Yinli Cai, Jiayu Zhao, Shisheng Sun

**Affiliations:** ^1^ Laboratory for Disease Glycoproteomics College of Life Sciences Northwest University Xi'an 710069 P. R. China

**Keywords:** data mining, glycan structures, glycoproteomics, multi‐omics integration, thymic involution

## Abstract

Glycosylation plays an important role in regulating innate and adaptive immunity. With promising advances in structural and site‐specific glycoproteomics, how to thoroughly extract important information from these multi‐dimensional data has become another unresolved issue. The present study reports a comprehensive data mining strategy to systematically extract overall and altered glycan features from quantitative glycoproteome data. By applying the strategy to investigation of thymic involution, the study not only presents a high‐resolution glycoproteome map of the mouse thymus, displaying distinct glycan structure patterns among immune‐relevant cellular components, but also uncovers four major altered glycan features associated with thymic involution, including elevated LacdiNAc mainly on the MHC class I complex, increased sialoglycans that perform multiple immune functions, down‐regulated bisecting glycans mostly linked to a sole GlcNAc branch, as well as possible shifts of glycan structures at the same glycosites. Regulatory network analyses further reveal the coordinated interactions of altered glycans with upstream regulators, including glycosyltransferases, glycosidases, and glycan‐binding proteins, as well as downstream signaling pathways. These data offer valuable resources for future functional studies on glycosylation and the mechanistic investigation of thymic involution, supporting the strategy as a powerful tool for in‐depth mining of structural and site‐specific glycoproteome data from various biomedical samples.

## Graphical abstract

This study presents a comprehensive data mining strategy for in‐depth extraction of overall and altered glycan features from structural and site‐specific glycoproteome data across various biomedical samples. The strategy enables the uncovery of four major altered glycan features associated with thymic involution and reveals their coordinated interactions with upstream regulators and downstream signaling pathways.

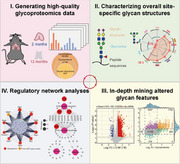

## 1 Introduction

Glycosylation, an important co‐/post‐translational modification of proteins, plays a key role in immune responses and signal transduction.^[^ ^1^ ^]^ Abnormal glycosylation has been linked to a multitude of diseases, including cancer, inflammation, and autoimmune diseases.^[^ ^2^ ^]^ Over the past decade, mass spectrometry‐based glycoproteomics has witnessed substantial advancements.^[^ ^3^, ^4^ ^]^ In particular, many search engines have been established for direct intact glycopeptide analyses, including Byonic,^[^ ^5^ ^]^ GPQuest,^[^ ^6^ ^]^ MSFragger‐Glyco,^[^ ^7^ ^]^ *O*‐Pair search,^[^ ^8^ ^]^ StrucGP,^[^ ^9^ ^]^ pGlyco3,^[^ ^10^ ^]^ Glyco‐Decipher,^[^ ^11^ ^]^ and PEAKS GlycanFinder.^[^ ^12^ ^]^ These sophisticated tools have empowered researchers to elucidate the composition or structural characteristics of glycans at each glycosite. Among them, our StrucGP software uniquely employs a modular strategy to precisely resolve the intricate structures of *N*‐glycans at each glycosite,^[^ ^9^ ^]^ enabling us to generate high‐resolution glycoproteomic data that contain a large amount of information on glycoproteins and multidimensional structural features of glycans. However, it remains challenging to thoroughly extract all important glycan structural features and recognize hub glycans and glycoproteins from these complex omics data.

Glycoproteins have long been recognized as important biomolecules involved in innate and adaptive immune responses.^[^ ^13^ ^]^ Glycosylation regulates the maturation, activation, and migration of immune cells to immune response sites,^[^ ^14^ ^]^ and dysregulation of glycosylation plays an essential role in the aging process and the development of age‐related diseases.^[^ ^15^, ^16^ ^]^ The thymus, the primary lymphoid organ for T cell proliferation, differentiation, and maturation, is the first organ to undergo age‐related involution.^[^ ^17^ ^]^ As an essential cause of immune senescence,^[^ ^18^ ^]^ thymic involution is mainly reflected by the reduction of thymic tissue mass and cell number, a blurring of boundaries between cortex and medulla, as well as morphological and structural disorders.^[^ ^19^ ^]^ The cortical and medullary regions of the thymus decrease in size and are subsequently replaced by adipose tissue.^[^ ^20^ ^]^ In addition, the proportion of memory T cells increases, along with a decline output of naive T cells and compromised T cell‐mediated immune function.^[^ ^21^ ^]^ These changes contribute to dysfunction of the immune system, which may lead to various diseases such as autoimmune diseases, cancers, and neurodegenerative diseases, as well as increased susceptibility to COVID‐19.^[^ ^22^ ^]^

Recent thymic involution studies have focused on transcriptomics with the help of single‐cell RNA sequencing, such as thymic stromal cells (TECs), coordinated T‐cell differentiation, and senescence‐related thymic dysfunction in the context of autoimmunity and immunodeficiency.^[^ ^23^, ^24^, ^25^ ^]^ Nevertheless, it is essential to also consider changes in proteins and their post‐translational modifications, especially glycosylation, to gain a comprehensive understanding of the aging process.^[^ ^26^ ^]^ To date, the glycosylation information of thymic tissues, thymocytes, and thymic microenvironment has been mainly obtained using the lectin and immunohistochemical methods,^[^ ^27^, ^28^ ^]^ which are incapable of detecting the detailed glycan structures as well as their corresponding glycosites and glycoproteins.

In this study, we propose a comprehensive strategy for glycoproteome data mining and apply it to glycoproteomic analysis of mouse thymic involution. We first obtained high‐quality glycoproteomic data by combining the TMT label quantification, offline fractionation, mass spectrometry parameter optimization, and StrucGP‐based site‐specific glycan structure interpretation. We then characterized the overall profile of site‐specific glycans in mouse thymus based on the overview of glycoproteins and glycans, their detailed sub‐structural features, as well as distinct glycan structure patterns associated with various subcellular locations or biological processes. We next systematically extracted the altered glycan structure features from these quantitative glycoproteomic data across various aspects, including the top 10 up‐ and down‐regulated glycans, overall as well as uniquely up‐ and down‐regulated glycan features, the glycan sub‐structures changed at a high proportion or high fold‐change, the normalized glycosylation changes by eliminating their protein expression changes, the possible shift of glycan structures at the same glycosites across different age stages, as well as functionally essential glycopeptides. Finally, we also performed regulatory network analyses via multi‐omics data to further acquire the upstream and downstream information associated with altered site‐specific glycans, including the glycosyltransferases, glycosidases, glycan‐binding proteins, the related signaling pathways, and interaction networks. In addition to the distinct glycan structure patterns associated with immune‐related cellular components or biological processes, this data mining strategy also allowed us to find four major glycan structure alterations during mouse thymic involution, as well as the cooperative changes in glycosyltransferase, glycan‐binding proteins, and signaling pathways. All these findings provide valuable resources for future studies on important glycosylation functions and possible mechanisms of thymic involution, highlighting the importance of this data mining strategy in advancing comprehensive glycoproteomic analyses across a variety of biomedical samples.

## 2 Results

### 2.1 A Comprehensive Data Mining Strategy for High‐Resolution Glycoproteomics

**Figure 1:**
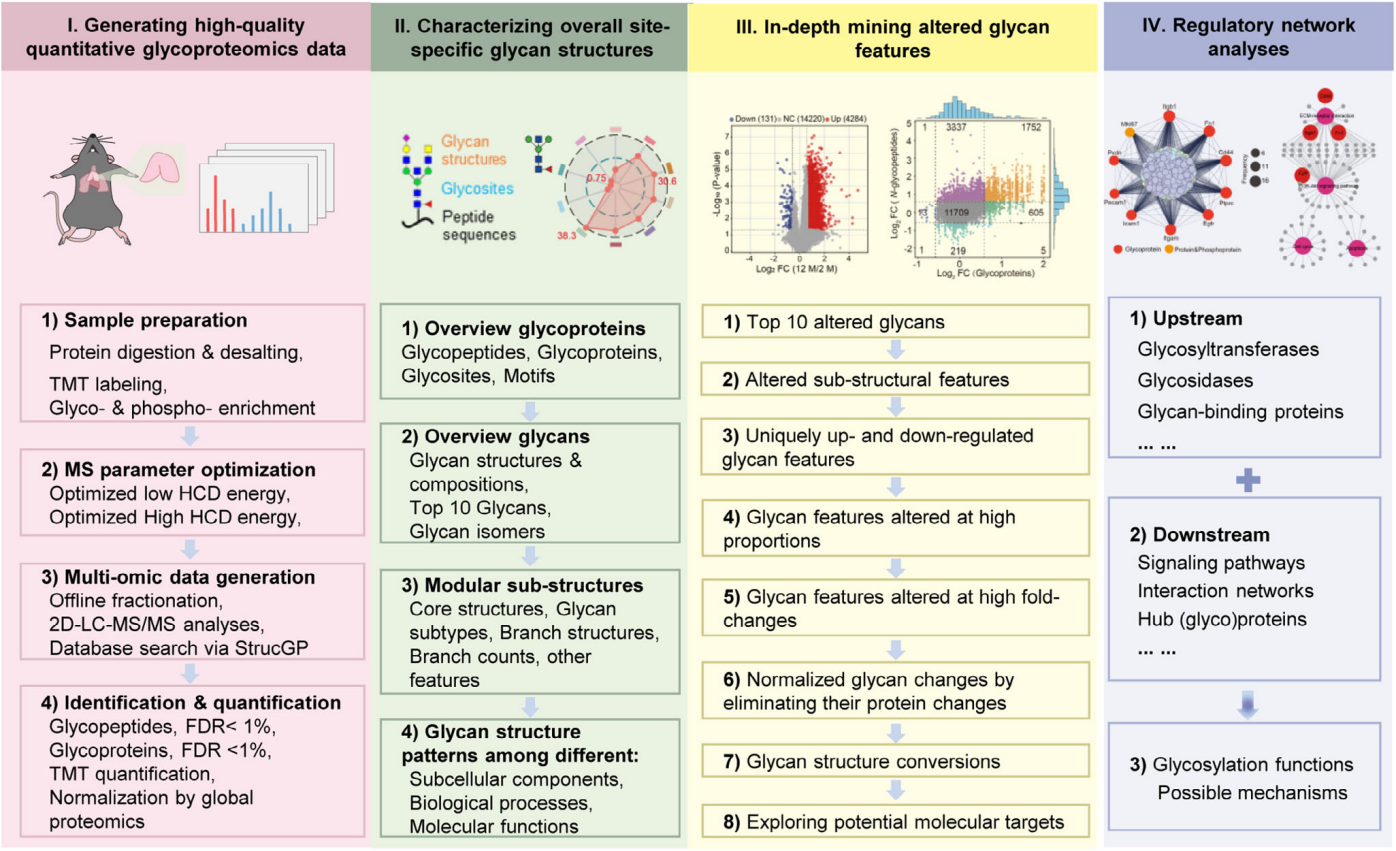
Workflow for high‐quality *N*‐glycoproteomics data mining. The whole workflow consists of four major components: I) Generating high‐quality quantitative glycoproteomics data; II) Characterizing overall site‐specific glycan structures; III) In‐depth mining of altered glycan features; and IV) Regulatory network analyses using multi‐omics data.

In this study, we proposed a comprehensive data mining strategy for extracting qualitative and quantitative glycan structure features from high‐resolution *N*‐glycoproteomic data (**Figure** **1**; Note S1, Supporting Information). The entire workflow consists of four major components: (I) Generating high‐quality quantitative glycoproteomics data; (II) Characterizing overall site‐specific glycan structures; (III) In‐depth mining of altered glycan features; and (IV) Regulatory network construction using multi‐omics data. This data mining strategy was applied to explore structural alterations of site‐specific *N*‐glycans during thymic involution.

To obtain high‐quality quantitative glycoproteomic data, we used tandem mass tag (TMT) labeling to ensure the quantification accuracy of intact glycopeptides (IGPs), and the mass spectrometry parameters were further optimized to achieve optimal identification and quantification. The IGPs were also fractionated offline before liquid chromatography–tandem mass spectrometry (LC‐MS/MS) analyses to further increase the glycopeptide coverage. By using our StrucGP‐based approach, we quantitatively identified a large number of detailed *N*‐glycan structures at the site‐specific level, as well as many additional *N*‐glycosites and glycoproteins within FDR<1% at the glycosite‐containing peptide level. In addition, we also used the factors from the global proteome to normalize glycopeptides to ensure their consistent and accurate quantification. These steps ensured the generation of in‐depth and high‐quality glycoproteome data from various biomedical samples.

The profile of high‐resolution glycoproteome was presented in four major aspects: i) Overview of glycoproteins, including identified glycoproteins and glycosites, the distribution of glycosites on each glycoprotein as well as their motifs; ii) Overview of glycans, including the total glycan structures and compositions (showing the overall complexity of *N*‐glycans), the top 10 glycans (a snapshot of *N*‐glycans), and structural isomers of each glycan composition or glycosite. iii) Detailed structural features of site‐specific *N*‐glycans. Based on our modular strategy,^[^ ^9^ ^]^ *N*‐glycans could be further separated into core structures, glycan subtypes, branch structures, and branch counts, as well as other structural features such as different types of fucosylation or sialylation. The distributions of these sub‐structures could give us a better understanding of the glycosylation features of the experimental samples. iv) Glycan structure patterns associated with various subcellular locations or biological processes. By locating site‐specific glycans or structural features in different cellular components or biological processes via their attached glycoproteins, we could uncover distinct glycan patterns among different cellular components or biological processes. All the above information provided a general outline of the high‐resolution *N*‐glycoproteomic landscape in experimental samples.

Altered glycan structure features were extracted across the following aspects: i) Top 10 up‐ and down‐regulated glycans were used to show a general outline of altered glycans. ii) Altered glycan features, including core structures, glycan subtypes, branch structures, and other structural features, were systematically analyzed as described above. iii) Uniquely up‐ and down‐regulated glycan features were determined based on their proportional differences between up‐ and down‐regulated IGPs. iv) Highly altered glycan features were extracted based on their proportions within the glycopeptides that contained the sub‐structures. v) Glycan features enriched in high fold‐change ranges. vi) Normalized glycosylation changes by eliminating their protein expression changes (integrated with quantitative proteomic data). vii) Possible shift of glycan structures at the same glycosites among different samples or conditions. viii) Important glycopeptides, which might be involved in some important signaling pathways and biological processes, are located at some important cellular components, or have a high fold‐change. These specific glycoproteins or glycans have great potential to serve as important biomarkers or therapeutic targets.

Regulatory network analyses through multi‐omics data could provide additional insights into the changes in upstream glycosylation regulators (such as glycosyltransferases, glycosidases, and glycan‐binding proteins), and downstream signaling pathways that were associated with altered site‐specific glycans. Proteomic data could also be used to eliminate the glycopeptide changes that occurred at their protein expression levels. These analyses offer valuable up‐ and down‐stream information on altered site‐specific glycans, enabling the exploration of potential glycosylation functions and mechanisms involved in various physiological and pathological conditions.

### 2.2 Generation of High‐Quality Quantitative *N*‐Glycoproteomics Data

**Figure 2:**
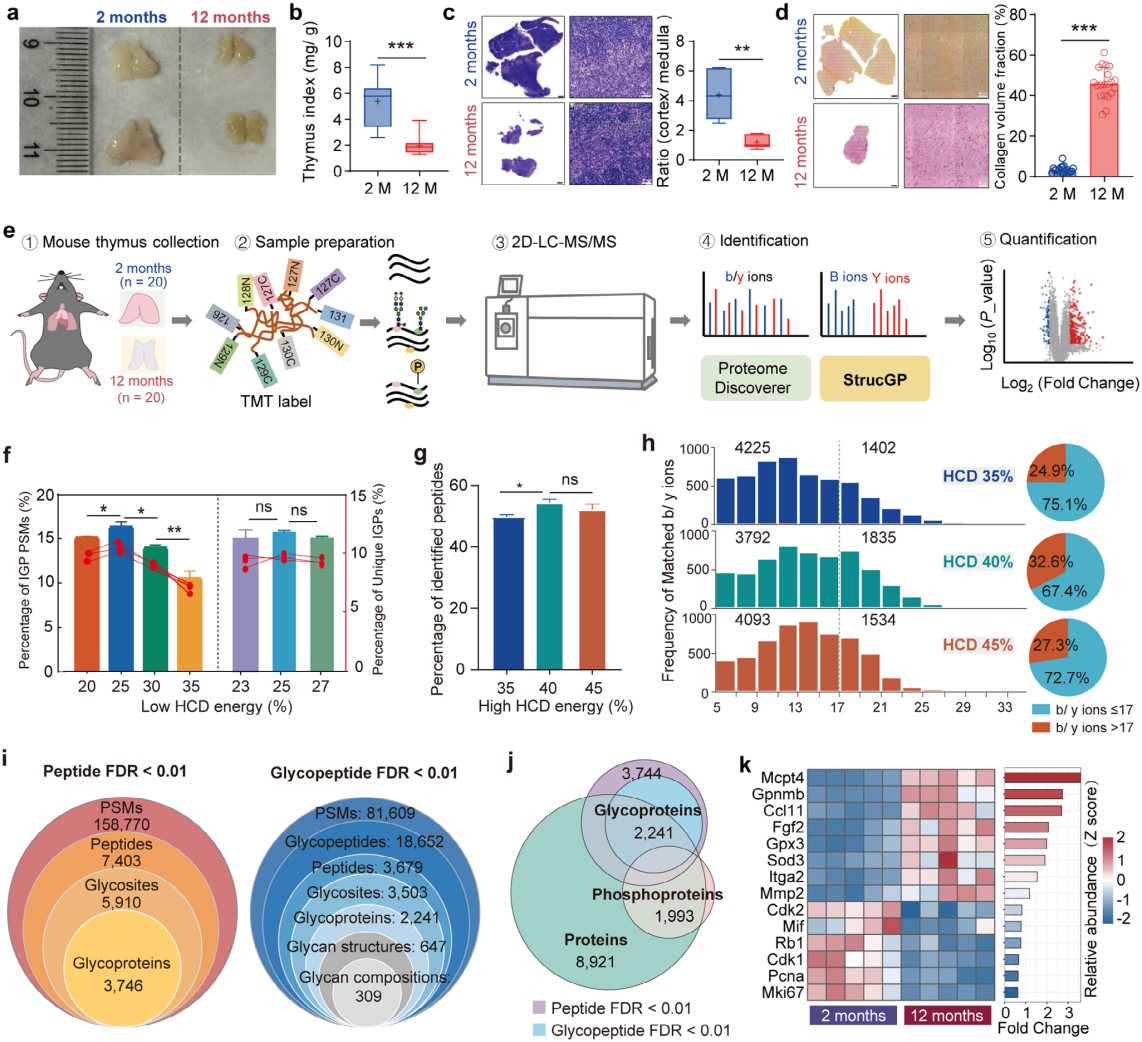
Generating high‐quality glycoproteomic data associated with age‐related thymic involution in mice. a) Morphological images of thymus tissues from 2‐ to 12‐month‐old mice. b) Thymus index of mice in each age group. The index indicates the ratio between the thymus and the mouse body weights. Data are presented as mean ± SD. *n* = 20, ^***^ *p* < 0.001 by Mann–Whitney *U* test. c) HE staining of mouse thymus (left panel, Scale bar, 500 µm) and the ratio of cortical to medullary area of mouse thymus in both age groups (right panel, *n* = 6). Data are presented as mean ± SD. ^**^ *p* < 0.01 by unpaired *t*‐tests. d) Sirius Red staining showing the collagen fiber deposition in the thymus (left panel, Scale bar, 500 µm) with statistical analyses (right panel, *n* = 20). Data are presented as mean ± SD. ^***^ *p* < 0.001 by unpaired *t*‐tests. e) Workflow for quantitative glyco‐, phospho‐, and global proteome analyses of thymus tissues from 2‐ to 12‐month‐old mice (6 males and 14 females per age group). f) Evaluation of different low HCD energies for interpreting glycan structures on TMT‐labeled glycopeptides. g) Percentages of identified glycosite‐containing peptides on TMT‐labeled glycopeptides using different high HCD energies. h) Distribution (left bar graphs) and percentages (right pie charts) of matched b/y ions for identifying glycosite‐containing peptides at different high HCD energies. i) Overall profile of intact glycopeptides, glycoproteins, glycosylation sites, glycan structures, and glycan compositions identified in both age groups (peptide FDR < 0.01, glycopeptide FDR < 0.01). j) Identification of proteins, glycoproteins, and phosphoproteins in mouse thymus. Identification of glycoproteins was controlled by FDR <1% at glycosite‐containing peptide level (the outer purple circle) or at the intact glycopeptide levels (the inner blue circle). k) Heat map of senescence‐related marker expressions detected by global proteomic data in this study. PSMs: peptide‐spectrum matches. TMT: tandem mass tag. FDR: false discovery rate. Hex: Hexose. HexNAc: N‐acetylhexosamine. Oxo: oxonium ions.

We applied the above data mining strategy to explore key site‐specific glycan alterations during thymic involution in mice. Thymus tissues were collected from young (2‐month‐old, *n* = 20) and middle‐aged mice (12‐month‐old, *n* = 20) (**Figure** **2**a), which approximately correspond to human ages of 15–18 and 42–45 years old, respectively.^[^ ^29^ ^]^ In comparison to young mice, the middle‐aged mice exhibited increased body weights but decreased thymus weights, and therefore a declined thymus index (Figure 2b; Figure S1a,b, Supporting Information). In addition, the middle‐aged thymus showed notable atrophy with a less discernible boundary, as well as a diminished ratio between cortex and medulla (Figure 2c). The middle‐aged thymus also exhibited pronounced fibrosis with elevated collagen (Figure 2d; Figure S1c, Supporting Information). All aforementioned data confirmed that middle‐aged mice underwent significant involution in the thymus and thus were suitable for subsequent thymic involution studies. The intricacies of site‐specific *N*‐glycosylation and its alterations during thymic involution were then investigated using the StrucGP‐based *N*‐glycoproteomic approach^[^ ^9^ ^]^ (Figure 2e). Specifically, twenty thymus samples from each age group were combined into five pooled samples, and proteins extracted from the pooled samples were digested into peptides before 10‐plex TMT labeling. Ten TMT‐labeled samples were combined to form a single pooled sample, and then a small portion was directly used for quantitative proteomic analyses, while the remaining portions underwent sequential enrichment of intact glycopeptides and phosphopeptides for glyco‐ and phospho‐proteome studies, respectively.

To ensure optimal identification and quantification, we systematically evaluated the performance of different HCD collision energies in characterizing these TMT‐labeled intact *N*‐glycopeptides at both glycosite‐containing peptide and glycan structure levels.^[^ ^9^ ^]^ Among four low HCD energies (20–35%) used for detailed glycan structure interpretation, HCD 25% achieved the best glycan structure identification (Figure 2f). By further evaluating HCD energies surrounding the HCD 25%, we found that the HCD 25 ± 2% could achieve similar glycan structure identification (Figure 2f). Among three high HCD energies (35%, 40%, 45%) used for peptide identification, the HCD 40% achieved slightly better peptide identification compared to the other two HCD energies (Figure 2g,h). Altogether, the HCD 40% and 27% were finally selected as two suitable HCD energies for determining the peptide sequences and *N*‐glycan structures of TMT‐labeled intact *N*‐glycopeptides, respectively.

To increase the depth and coverage of quantitative glyco‐, phospho‐, and global proteomic analyses, we further separated all glyco‐, phospho‐, and total peptide samples into 12 or 24 fractions before LC‐MS/MS. Using this optimized site‐specific and structural glycoproteomic approach, we identified 18652 unique IGPs from TMT‐labeled thymus samples within a 1% false discovery rate (FDR) at both glycosite‐containing peptide and glycan levels (Figure 2i). These thymic IGPs were comprised 647 *N*‐glycans and 3503 glycosite‐containing peptides from 2241 glycoproteins, which represented the most comprehensive glycoproteome map of the mouse thymus to date (Figure 2i,j; Table S1, Supporting Information). The numbers of identified *N*‐glycosites and glycoproteins were further increased to 5910 and 3746, respectively, when the 1% false discovery rate (FDR) was controlled only at the glycosite‐containing peptide level (Figure 2i). From phosphoproteomic and proteomic data, we also identified 1993 phosphoproteins and 8921 proteins, respectively (Figure 2j; Table S2, Supporting Information). Notably, a range of senescence‐associated markers were identified through quantitative proteomics analysis, thereby supporting the validity of the aging model. These included the upregulated secretory phenotype (SASP)‐related factors (Ccl11, Mcpt4, Fgf2, Itga2, Mmp2), reactive oxygen species (ROS)‐related proteins (Gpx3, Sod3), and non‐metastatic melanoma protein B (Gpnmb),^[^ ^30^, ^31^ ^]^ as well as downregulated proteins related to cell cycle and cell proliferation, such as Cdk1, Cdk2, Pcna, Mki67, and Rb1 (Figure 2k).

### 2.3 Overall Structural Characterization of Site‐Specific *N*‐Glycans

**Figure 3:**
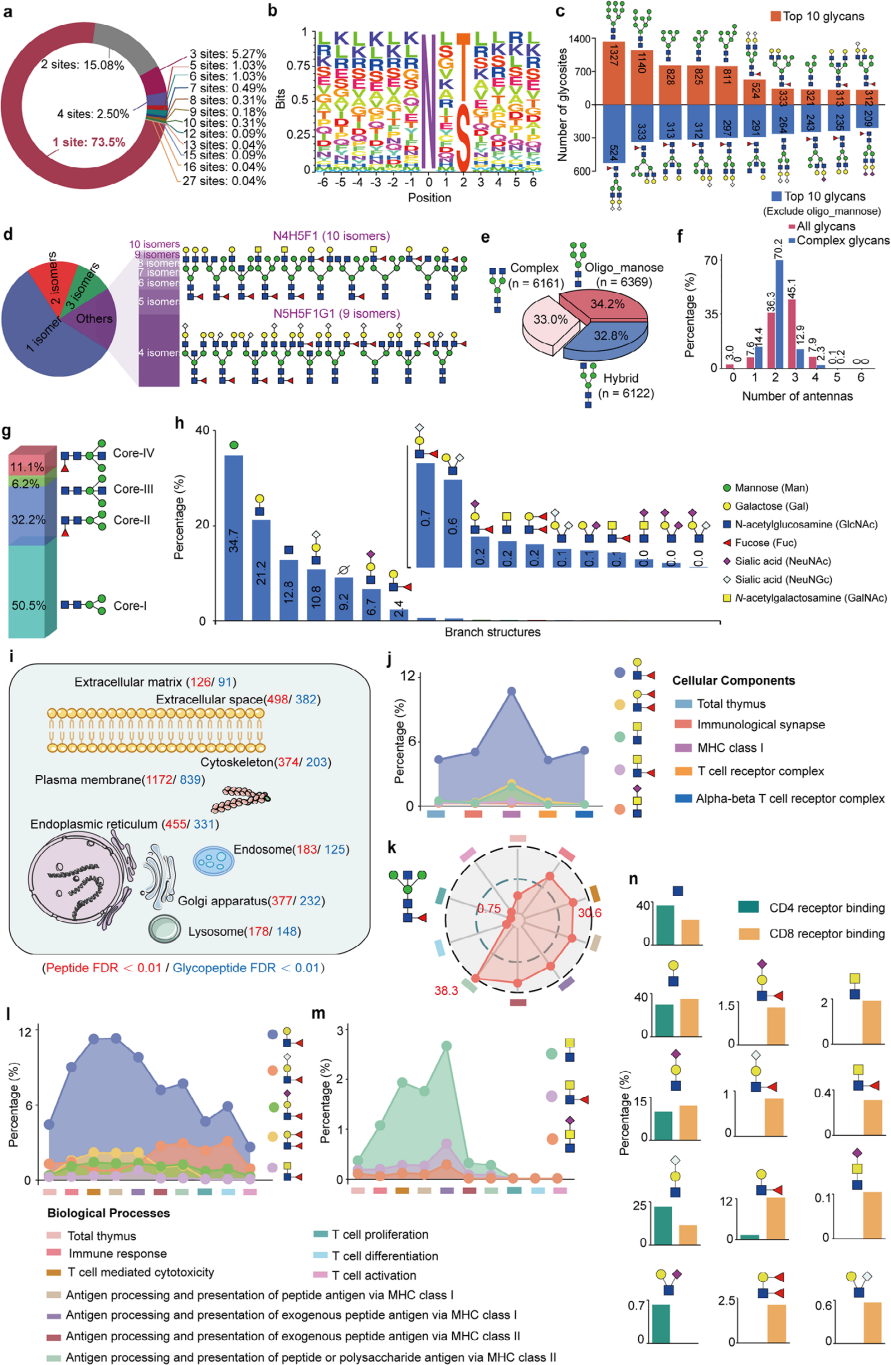
Overall characterization of site‐specific *N*‐glycans in mouse thymus. a) Number of glycosites identified on each glycoprotein. b) Glycosite motif analysis of glycoproteins identified at the glycopeptide FDR < 0.01 level in mouse thymus tissue. c) Top ten glycan structures detected in mouse thymus based on the number of their modified *N*‐glycosites.d) Proportions of glycan structure isomers identified in mouse thymus (pie chart, left panel), as well as two examples showing 9–10 different structural isomers discriminated from each glycan composition of the N4H5F1 and N5H5F1G1 (right panel). e) Proportions of different glycan subtypes based on unique glycopeptides. f) Distribution of branch counts on all glycans or only complex glycans. g,h) Proportions of core structures (g) and branch structures (h) in mouse thymus. The percentages were calculated based on the unique glycopeptides modified by each core or branch glycan structure. “⌀:” lack of one branch. i) Distribution of subcellular localization of mouse thymic glycoproteins. Red and blue represent mouse thymic glycoproteins identified with FDR < 0.1 at the glycosite‐containing peptides and the intact glycopeptides, respectively. j) Distribution of Lewis^a/b^ and LacdiNAc branch structures among glycoproteins located at different immune‐related cellular components. k–m) Abundance of core‐IV (k), branch fucose (l), and three LacdiNA‐containing branch structures (m) across different immune‐related biological processes. n) Branch structures that were specifically enriched in CD4 and/or CD8 receptor binding entries.

We first characterized the overall glycoproteomic features of the mouse thymus. The majority of glycoproteins (73.5%) contained one glycosite, although up to 31 *N*‐glycosites were identified from the glycoprotein Lrp1 (**Figure** **3**a; Figure S2a, Supporting Information). The motif analyses on these identified *N*‐glycosites showed a comparable proportion of N‐X‐S and N‐X‐T motifs (X represents amino acids other than proline) in mouse thymus (Figure 3b). The top 10 *N*‐glycans in mouse thymus (based on the numbers of modified glycosites) included 6 oligo‐mannose (Man4–Man9), 2 complex (N4H5F1G2 and N5H5F1), and 2 hybrid glycans (N4H7F1 and N4H6F1G1. N: HexNAc; H: Hex; F: Fucose; S: Neu5Ac; G: Neu5Gc). After excluding oligo‐mannose glycans, the remaining top 10 glycans were mainly core‐fucosylated (9/10) and/ or sialylated (6/10), with 7 complex and 3 hybrid glycans (Figure 3c). The 647 identified *N*‐glycan structures could be sorted into 309 different compositions, indicating that a large number of glycan compositions (45.1%) have been identified as distinct structural isoforms using the StrucGP software. Especially, the glycan compositions of N4H5F1 and N4H5F1G were further distinguished as 9–10 distinct glycan structures (Figure 3d), and up to five glycan isomers were detected at the same glycosite (Figure S2b, Supporting Information). These glycan isomers were distinguished from one another based on their feature B and Y ions at low HCD energy of MS/MS spectra (Figure S3, Supporting Information).

We then investigated the detailed sub‐structural features of site‐specific *N*‐glycans in mouse thymus by classifying these *N*‐glycans based on their core structures (4), branch structures (17), and/or glycan subtypes (3). Among all identified glycopeptides, 34.2% were modified by oligo‐mannose, followed by complex (33.0%) and hybrid glycans (32.8%) (Figure 3e). The majority of glycans were bi‐ and tri‐antennary, representing 36.3% and 45.1% of the total glycans, respectively. In addition, 70.2% of the complex glycans were bi‐antennary, and up to six antennas (only three IGPs) were identified from a single glycan (Figure 3f). It should be mentioned that mammalian glycans typically possess no more than five antennas,^[^ ^30^ ^]^ and therefore the glycans shown in Figure S4 (Supporting Information) might also be polyLacNAc structures, although these putative structures could not be confirmed due to the lack of essential feature B/Y ions. Among the four types of core structures, the most prevalent one was N2H3 (Core‐I), accounting for 50.5% of all unique IGPs. Other core structures included core‐fucosylated (Core‐II, 32.2%), bisected (Core‐III, 6.2%), and both (Core‐IV, 11.1%) (Figure 3g). Of the 17 branch structures, oligo‐mannose (labeled as Hex) and LacNAc (NH) accounted for 34.7% and 21.2% of the unique IGPs, respectively. Other branch structures included the sole GlcNAc (12.8%), sialylated LacNAc with Neu5Gc (10.8%) and Neu5Ac (6.7%), as well as Lewis^x/a^ (2.4%) and Lewis^y/b^ (0.2%). In addition, the LacdiNAc with/without sialylation and/or fucosylation, as well as some other branch structures, exhibited relatively low abundances (Figure 3h). Also, a large number of site‐specific glycans were fucosylated (24.9%, including both core‐ and antenna‐fucoses), sialylated (9.7%, including both Neu5Ac and Neu5Gc), or both (20.5%) (Figure S2c, Supporting Information).

We next compared glycan structure patterns among different cellular components. The Gene Ontology (GO) enrichment analyses showed that the glycoproteins identified in mouse thymus were distributed at various cellular components, such as the plasma membrane (850), extracellular space/ matrix (473), endoplasmic reticulum (337), and Golgi apparatus (243) (Figure 3i). Especially, many cellular components exhibited distinct glycan structure patterns. For instance, the branch structures containing LacdiNAc were more prevalent in the extracellular matrix, endoplasmic reticulum, and Golgi apparatus compared to other cellular components. The extracellular matrix glycoproteins exhibited a higher percentage of complex glycans and core‐fucosylation, whereas the glycoproteins in the endoplasmic reticulum displayed a high percentage of oligo‐mannose glycans and the normal core structure (Core‐I) (Figure S2d–f, Supporting Information). We paid special attention to the glycan structure features on various immune‐related components and found that the LacdiNAc structure and Lewis epitopes were highly enriched in the MHC class I complex compared to other immune‐relevant cellular components (Figure 3j; Figure S2g, Supporting Information). The Core‐IV, LacdiNAc structure and Lewis epitopes were enriched in the biological processes of the T cell‐mediated cytotoxicity and the immune response, but were rarely present in the T cell proliferation, activation, and differentiation (Figure 3k–m; Figure S2g, Supporting Information). Similarly, a higher proportion of LacdiNAc branch structures was also enriched in MHC I‐mediated antigen presentation in comparison to MHC II‐mediated antigen presentation. In addition, glycan structure differences were also observed in the MHC I and MHC II complexes binding, as well as in the CD8 and CD4 receptors binding based on their molecular functions (Figure 3n). It is widely acknowledged that the T‐cell antigen receptors (TCRs) and T‐cell membrane molecules (e.g., CD4 and CD8) on immune cells can specifically recognize the MHC molecules on antigen‐presenting cells to form an immunological synaptic structure,^[^ ^31^ ^]^ and it is therefore possible that the MHC I complexes and CD8 receptors binding exhibited a higher abundance of LacdiNAc compared to the MHC II complexes and CD4 receptors binding. The CD8 receptors binding also exhibited significant differences in the branch structures with antenna‐fucose, Core‐IV, and complex glycan subtype compared to CD4 receptors binding (Figure 3n; Figure S2g, Supporting Information).

### 2.4 In‐Depth Mining of Altered Glycan Features Associated with Age‐Related Thymic Involution

**Figure 4:**
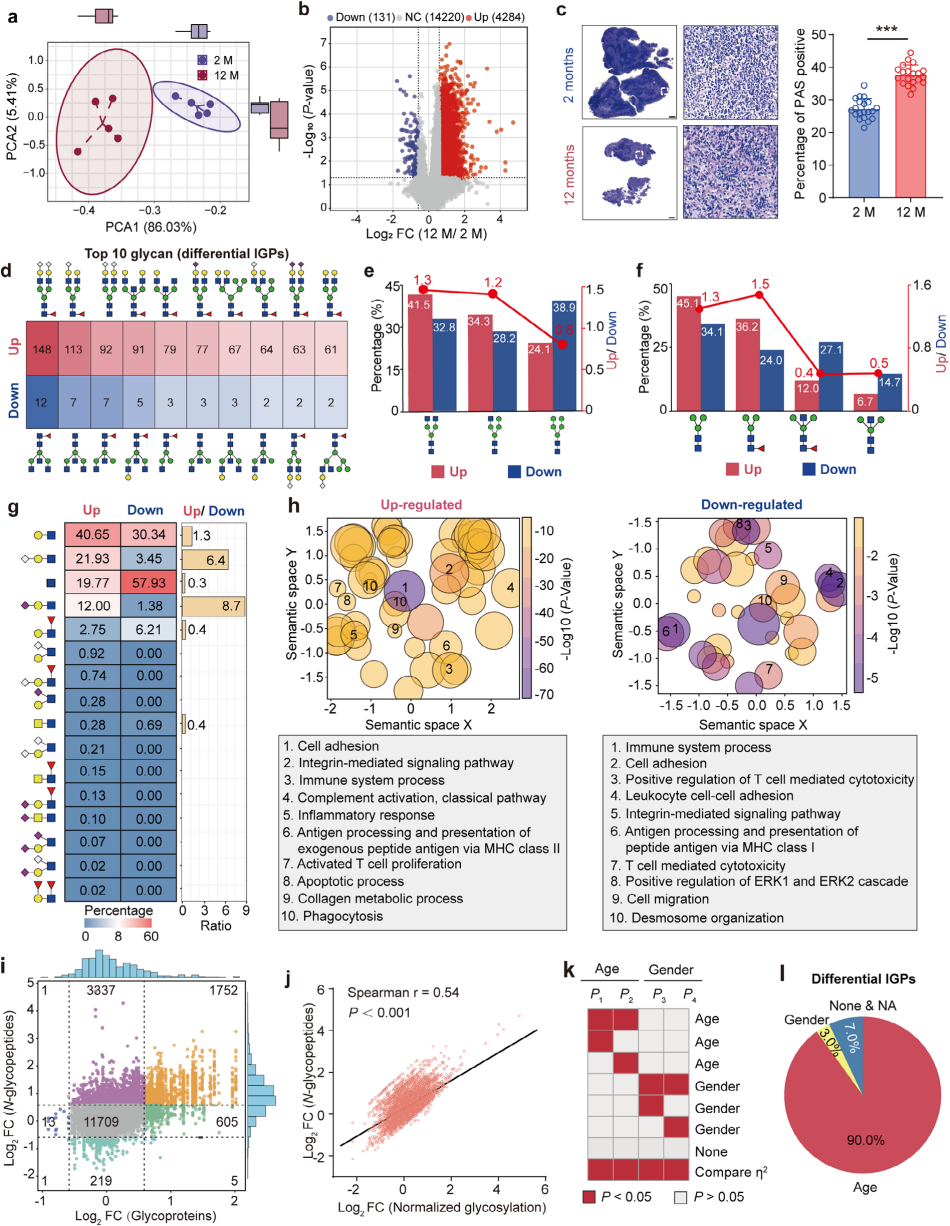
Site‐Specific *N*‐glycan changes of mouse thymic involution. a) Principal component analysis (PCA) plot showing distinct separation between young (2‐month‐old) and middle‐aged (12‐month‐old) thymic groups. b) Volcano plot highlighting significantly up‐ and down‐regulated *N*‐glycopeptides in the aged thymus. c) Periodic Acid Schiff (PAS)‐stained thymus sections from young and middle‐aged groups, visualizing glycosylation changes at the histological level (left panel, Scale bar, 500 µm), statistical analysis of PAS staining (right panel, *n* = 20). Data presented as mean ± SD. ^***^ *p* < 0.001 by unpaired *t*‐tests. d) Top 10 up‐ and down‐regulated site‐specific glycans (excluding the oligo‐mannose glycans). e–g) The proportions of different glycan subtypes (e), core structures (f), and branch structures (g) in up‐ and down‐regulated glycopeptides. The red dots represent the ratios of up‐regulated to down‐regulated glycan sub‐structures. h) Representative Gene Ontology (GO) biological process terms associated with up‐ and down‐regulated glycopeptides. Visualization of GO enrichment terms for differentially glycosylated proteins was performed using GO‐Figure. Each circle represents a GO term. The size of the circle corresponds to the number of genes associated with the term, the color scale indicates statistical significance (*p*‐value), and the spatial arrangement of the circles reflects semantic similarity between GO terms‐closer terms are more functionally related. i) Scatter plot depicting the differences in *N*‐glycopeptides and their corresponding glycoproteins in the aged thymus. j) Spearman correlation analysis (*r* = 0.54, *p* <0.001) of quantitative *N*‐glycopeptides before and after normalization to glycoproteins. k) One‐way ANOVA procedure for age and gender factors. Combined *p*‐values and effect sizes (η^2^) were used to assess their impact on the *N*‐glycoproteome. Significant features (*p* < 0.05) are shown in red; others are shown in grey. l) Pie chart showing the results of one‐way ANOVA analyses for differential IGPs.

We systematically investigated glycopeptide differences between young and middle‐aged mice based on TMT‐labeled quantification. The principal component analysis (PCA) indicated that considerable glycopeptide differences existed between the two age groups, with PC1 explaining 86% of the differences (**Figure** **4**a). By using the *p* < 0.05 and FC > 1.5 as a reasonable cutoff, we detected 4284 IGPs increased but only 131 decreased IGPs in the middle‐aged thymus (Figure 4b; Table S3, Supporting Information). The overall up‐regulated *N*‐glycosylation in the middle‐aged thymus was further confirmed using the Periodic Acid‐Schiff (PAS) staining (Figure 4c). Except for oligo‐mannose glycans (Man2–Man11), the top 10 up‐regulated site‐specific glycans were predominantly core‐fucosylated (90%) or terminated by sialic acids (60%, HexNAc+Hex+Neu5Gc/Neu5Ac), whereas the top 10 down‐regulated site‐specific glycans were mainly bisected GlcNAc (70%) and/or sole GlcNAc branch (70%) (Figure 4d). Based on the ratio of differential to total IGPs with each glycan substructure, we found that sialoglycans and LacdiNAc were significantly altered (Figure S5a–c, Supporting Information). As the numbers of up‐ and down‐regulated glycopeptides were also significantly different, we also calculated the percentage of glycan sub‐structures in up‐ and down‐regulated glycopeptides separately and further compared the up‐ and down‐regulated proportions of each sub‐structure. The substructural features of up‐regulated glycans (except oligo‐mannose glycans) were predominantly core‐fucosylated or sialyl‐LacNAc, whereas down‐regulated glycans were mainly bisected core (Core‐III & IV, 41.8%) and the sole GlcNAc branch (57.9%) (Figure 4e–g). When the cutoff was switched to FC > 2 and *p* < 0.05, the proportions of down‐regulated glycopeptides containing Core‐III & IV and the sole GlcNAc branch structure were further increased to 48.7% and 71.7%, respectively (Figure S5d,e, Supporting Information).

Biological processes enrichment analyses showed that glycoproteins with up‐regulated glycopeptides were mainly involved in cell adhesion, integrin‐mediated signaling, immune system processes, and complement activation, while glycoproteins with down‐regulated glycopeptides were closely associated with T‐cell‐mediated cell cytotoxicity, positive regulation of ERK1 and ERK2 cascades, and cell migration (Figure 4h). As some up‐ and down‐regulated glycopeptides originated from the same glycoproteins and were enriched in the same biological processes, we also focused on the conversions in the glycan structure of up‐ and down‐regulated IGPs on the same glycoproteins or glycosites (the details are shown below). In addition, we also identified many glycopeptides with relatively high fold changes, such as 38 differential IGPs with FC > 5. Especially, these greatly altered IGPs only belonged to several glycoproteins, including the immunoglobulin family, Nmt1, and Cpa3, implying the great value of these IGPs in additional function and mechanism studies (Figure S5g,h, Supportingation).

To further determine whether these glycopeptide changes occurred at the protein expression or glycosylation level, we performed integrated analyses with proteomic data. Compared with 23.7% of altered IGPs (4415/18652), only 3.9% of quantifiable proteins (349/8837) were changed in the middle‐aged thymus (FC > 1.5, *p* < 0.05) (Figure 4b; Figure S10b,c, Supporting Information). Among all differential glycopeptides, 80.7% had at least 1.5‐fold change at the glycosylation level, even after deducting their protein expression alterations (Figure 4i; Figure S5i,j, Supporting Information). That may explain why a relatively high degree of Spearman correlation (*r* = 0.54, *p* < 0.001) existed between the fold change ratios of glycopeptides with and without normalization using the related protein expression (Figure 4j). In addition, we also evaluated the effect of gender on glycopeptide changes within two age groups based on one‐way ANOVA analysis as described in the Methods section (Figure 4k). In order to examine the impact of age (or sex) on intact glycopeptides, we initially excluded the effect of sex (or age) and then assessed the differences between female and male mice (or between the two age groups). Using this strategy, we found that 3975 of 4415 differential IGPs (90.0%) were predominantly influenced by the age factor, whereas only 130 (3.0%) exhibited a strong correlation with the gender factor (Figure 4l), suggesting that the age factor was the primary determinant of differential glycosylation during thymic involution.

### 2.5 Major Glycan Alterations with Thymic Involution

Based on the above data mining strategy, we observed four major glycan structure alterations during age‐related thymic involution in mice.

#### 2.5.1 Elevated LacdiNAc Glycans Enriched in MHC Class I Protein Complex

**Figure 5:**
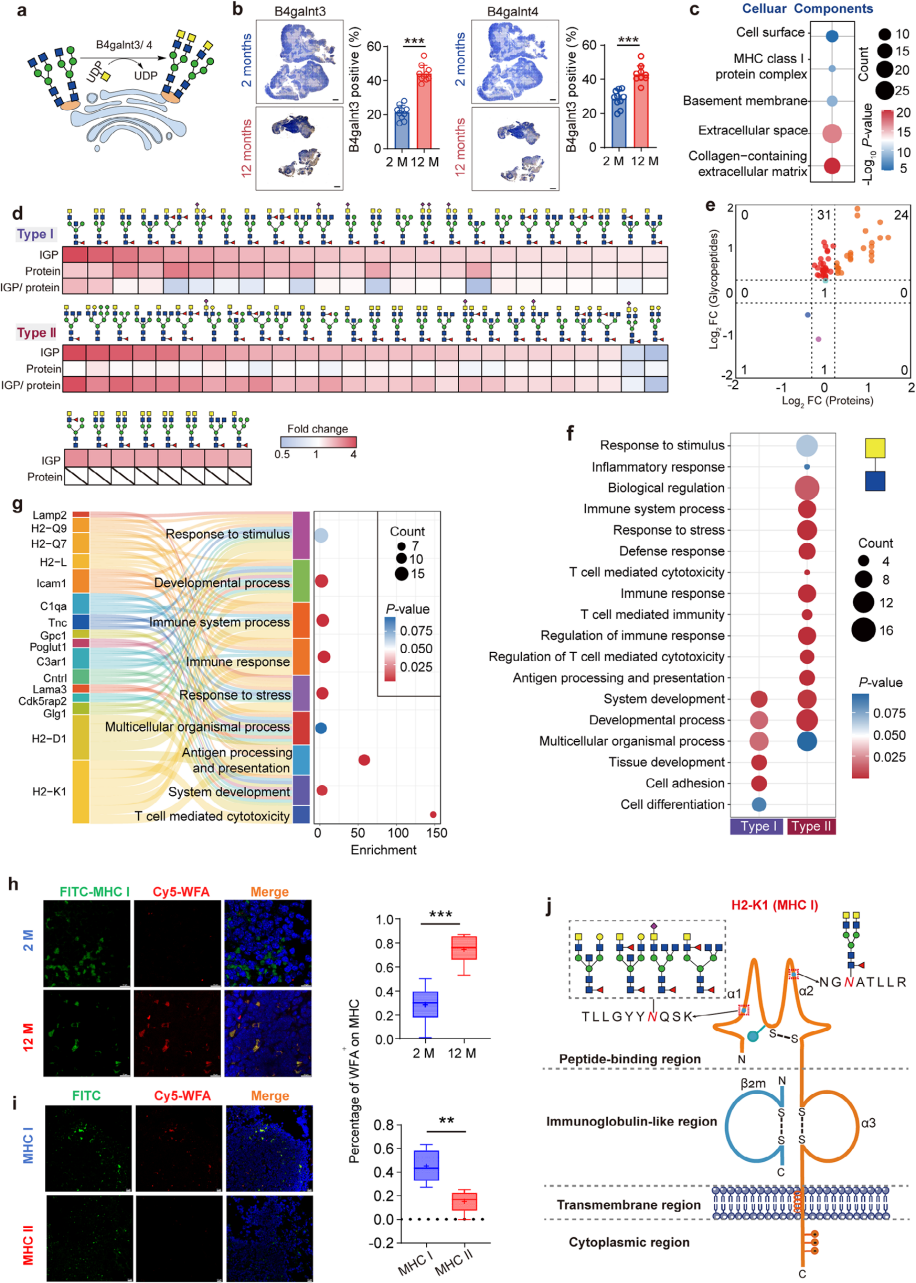
Elevated LacdiNAc glycans in middle‐aged mouse thymus. a) Schematic of LacdiNAc synthesis pathways. b) Immunohistochemistry of B4galnt3 and B4galnt4 protein expressions in mouse thymus tissues (Scale bar, 500 µm), statistical analysis of IHC (*n* = 10). Data presented as mean ± SD. ^***^ *p* < 0.001 by unpaired *t*‐tests. c) Subcellular localization of all identified glycoproteins containing LacdiNAc glycans. d) Heatmap of all quantitative LacdiNAc‐containing *N*‐glycopeptides showing the fold changes between young and middle‐aged mouse thymus tissues. e) Four‐quadrant volcano plots to show the changes of LacdiNAc‐containing *N*‐glycopeptides at the glycosylation and/or protein expression levels. f) Biological processes involved by glycoproteins with LacdiNAc‐containing glycopeptides changed at the protein expression (Type I) or glycosylation levels (Type II). Bubble size represents the number of enriched proteins per GO term, and color intensity reflects the statistical significance (*p*‐value) of enrichment. g) Sankey diagram highlighting representative gene ontology terms for type II LacdiNAc‐containing glycoproteins. The diagram further illustrates the major glycoproteins involved in selected representative GO terms, providing insights into the biological functions of type II LacdiNAc‐modified proteins. h) Lectin staining of LacdiNAc on MHC‐I in thymic sections from 2‐ and 12‐month‐old mice. WFA was used to detect LacdiNAc, and MHC‐I was labeled with a specific antibody. WFA‐positive cells were quantified (*n* = 10). Scale bar, 10 µm. Data are mean ± SD; unpaired two‐tailed *t*‐test, *p* < 0.05. i) Lectin staining of LacdiNAc on MHC‐I and MHC‐II in 12‐month‐old mouse thymus. WFA‐positive cells were quantified (*n* = 5). Scale bar, 10 µm. Data are mean ± SD; unpaired two‐tailed *t*‐test, *p* < 0.05. j) Up‐regulated LacdiNAc‐glycopeptides on H2‐K1 protein of the MHC I molecules. Glycosylation sites Asn‐107 and Asn‐197 are located in the α1 and α2 domains, which form the peptide‐binding region.

LacdiNAc, also known as LDN (GalNAcβ1‐4GlcNAc), was largely elevated during thymic involution (Figure 4g; Figure S4a–c, Supporting Information). Based on immunohistochemical (IHC) results, both related glycosyltransferases B4galnt3 and B4galnt4 were also up‐regulated in the middle‐aged thymus (**Figure** **5**a,b). In mouse thymus, we identified 139 LacdiNAc glycopeptides (including those with sialylation and/or fucosylation) from 52 glycoproteins. These LacdiNAc glycoproteins were mainly located at the collagen‐containing extracellular matrix, basement membrane, and MHC class I protein complex (Figure 5c). Among these glycopeptides with LacdiNAc glycans, 58 (41.7%) were significantly changed with thymic involution (*p* < 0.05), including 24 increased at protein expression levels (labeled as Type I), as well as 32 upregulated and 2 downregulated at the glycosylation level (labeled as Type II) (Figure 5d,e). The Type I glycoproteins were predominantly located in the collagen‐containing extracellular matrix and basement membrane and were involved in cell adhesion and tissue development. In contrast, the Type II glycoproteins were predominantly located in the MHC class I protein complex and the extracellular space and were closely associated with a range of biological functions, including antigen processing and presentation, immune system processes, and T cell‐mediated cell cytotoxicity (Figure 5f; Figure S6, Supporting Information). Further protein‐pathway interaction analyses revealed that the type II glycoproteins were primarily a series of MHC class I molecules, including H2‐Q9, H2‐K1, H2‐D1, H2‐L, and so forth (Figure 5g). The uniquely upregulated LacdiNAc in MHC‐I during thymic involution was further confirmed by the lectin histochemistry via Wisteria Floribunda Lectin (WFA) (Figure 5h). Notably, the lectin histochemistry results also showed that the MHC class II molecules exhibited minimal presence of LacdiNAc structures compared to the MHC class I molecules (Figure 5i), further supporting our observation that LacdiNAc glycans were preferentially enriched on MHC class I glycoproteins (Figure 3n). It is established that the MHC I molecules comprise an α‐chain and a β‐chain through non‐covalent binding. The α1 and α2 regions constitute the peptide‐binding region, while the α3 region binds to the CD8 molecules on the surface of T cells.^[^ ^32^ ^]^ In particular, we identified two glycosites in the H2‐K1 that contained the LacdiNAc glycans, including the glycosite Asn‐107 located at the α1 region and the glycosite Asn‐197 located at the α2 region (Figure 5j). These findings suggest that LacdiNAc glycans on MHC I molecules may be involved in antigen processing and presentation, as well as other related immune responses.

#### 2.5.2 Increased Sialoglycans Associated with Immune‐Related Functions

**Figure 6:**
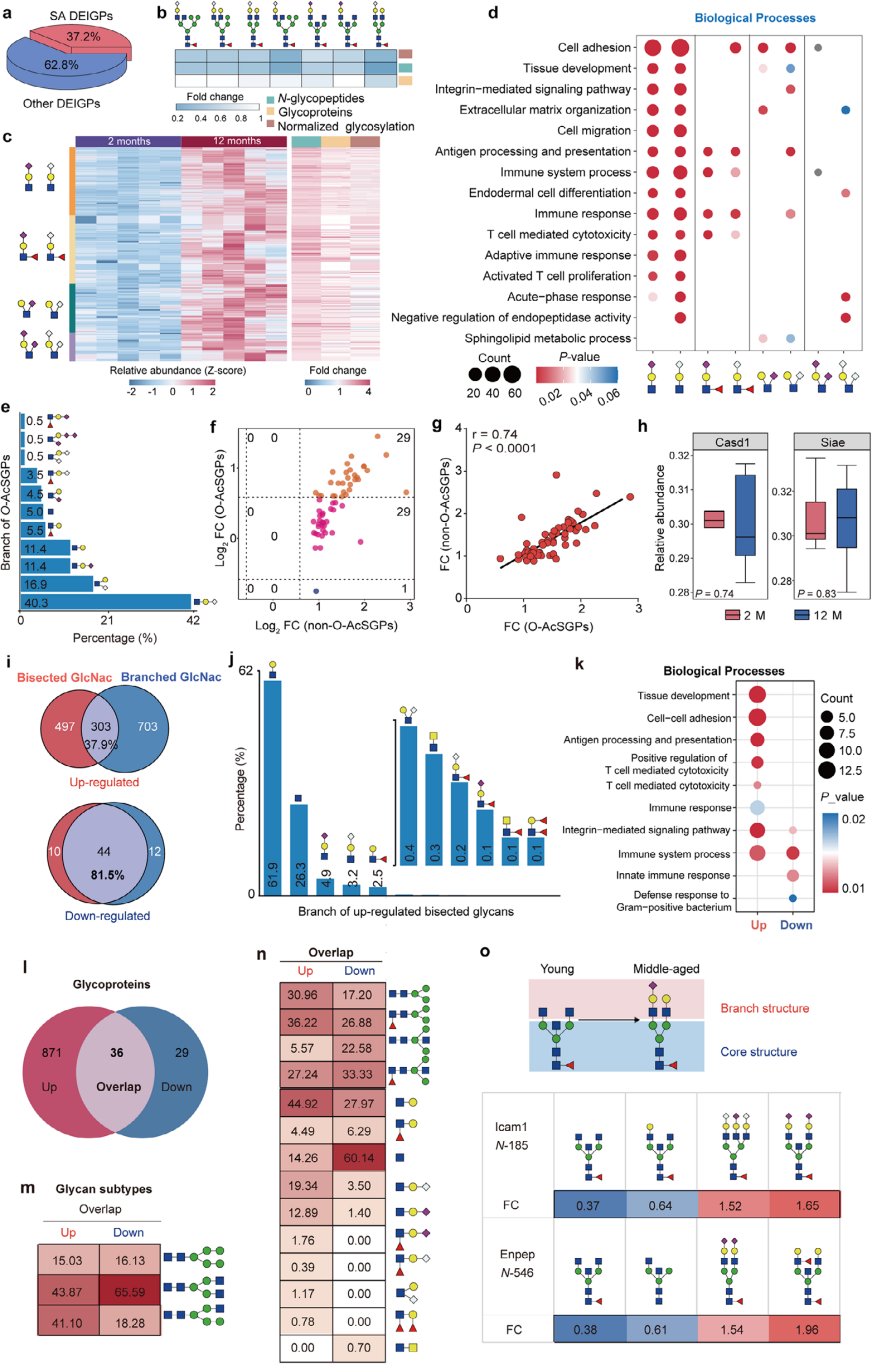
Analyses of increased sialoglycans, decreased bisected glycans, and their conversions during mouse thymic involution. a) Differential sialoglycopeptides account for more than one‐third of all differential glycopeptides. DEIGP: differentially expressed intact glycopeptides, SA: sialic acid. b,c) Heatmap of down‐ (b) and up‐regulated (c) sialoglycopeptides during mouse thymic involution, indicating that a large number of sialylated *N*‐glycopeptides were changed at the glycosylation level instead of protein expression level in the middle‐aged mouse thymus. d) Representative biological process terms involved by the four types of altered sialoglycopeptides. e) Abundance of 11 branch structures identified in *O*‐AcSGPs from mouse thymus. *O*‐AcSGPs: O‐acetylated sialoglycopeptides. f) Four‐quadrant diagram shows the expression of *O*‐AcSGPs and their corresponding non‐*O*‐acetylated forms of sialoglycopeptides. g) Correlation analyses for fold changes of *O*‐AcSGPs and their corresponding non‐*O*‐acetylated forms in mouse thymic involution (spearman *r* = 0.741, *p* <0.001). h) Boxplot shows the expression of *O*‐acetyltransferases Cisd1 and sialic acid esterases Siae. *n* = 5 per group. *p*‐values were calculated using unpaired *t*‐tests. i) Proportions of up‐ and down‐regulated glycopeptides containing both bisected and branched GlcNAc glycopeptides. j) Proportions of different branch structures in up‐regulated bisecting site‐specific glycans. k) Representative biological process terms for up‐ and down‐regulated bisected *N*‐glycopeptides. l) Venn diagram shows up‐ and down‐regulated glycopeptides corresponding to glycoproteins, and the overlap shows that many up‐ and down‐regulated glycopeptides originate from the same glycoproteins. m,n) Distribution of different glycan subtypes (m), core structures, and branch structures (n) among up‐ and down‐regulated glycopeptides occurred at the same glycoproteins. o) Demonstration of up‐ and down‐regulated glycan structures conversion at the same glycosite.

Sialylated glycopeptides modified by *N*‐glycolylneuraminic acid (Neu5Gc) and/or *N*‐acetylneuraminic acid (Neu5Ac) accounted for 37.2% of all differential glycopeptides (**Figure** **6**a). Except for seven decreased sialylated glycopeptides, 67.4% (1287/1896) of sialylated glycopeptides increased at the glycosylation level rather than the protein expression level during thymic involution. (Figure 6b,c; Figure S7a, Supporting Information). These dysregulated sialylated glycoproteins were predominantly enriched in immune‐related processes, such as the immune system process, defense response, inflammatory responses, and regulation of immune response. Additionally, they were implicated in cell adhesion, extracellular matrix assembly, and leukocyte migration (Figure 6d). It is known that the N‐acetyl group in Neu5Ac is reduced to N‐hydroxyethyl by cytidine monophosphate N‐acetylneuraminic acid hydroxylase (CMAH) in the cytoplasm, resulting in the conversion of CMP‐Neu5Ac to CMP‐Neu5Gc (Figure S7b). Subsequent functional analyses demonstrated that sialylated glycoproteins modified by Neu5Ac and/or Neu5Gc exhibited analogous biological functions (Figure S7c,d), but glycoproteins modified by different types of sialylated branch structures preferred to perform distinct biological functions (Figure 6d). For example, glycoproteins modified by sialylated LacNAc were involved in the biological processes of cell adhesion, tissue development, cell migration, immune response, and antigen presentation. While glycoproteins modified by sLewis^x/a^ were primarily associated with immune‐related biological processes (Figure 6d). We also investigated the *O*‐acetylated sialoglycopeptides (*O*‐AcSGPs) using our previously reported method^[^ ^33^ ^]^ (Figure S7e,f, Supporting Information), which revealed that *O*‐acetylation predominantly occurred at the Neu5Gc residues of sialyl‐LacNAc (Figure 6e). Increased *O*‐AcSGPs were also largely observed during thymic involution (Figure 6f), and were predominantly located in the extracellular space or extracellular matrix, performing the negative modulation of peptidase and endopeptidase activities (Figure S7g, Supporting Information). Further analyses showed that their alterations were positively correlated with the alterations of their non‐*O*‐acetylated forms (*r* = 0.74, *p* < 0.001, Figure 6g), and no significant change was observed in the related *O*‐acetyltransferase Cisd1 and sialyltransferase Siae (Figure 6h), implying that the up‐regulation of *O*‐AcSGP might be mainly caused by the overall up‐regulated sialoglycans.

#### 2.5.3 Decreased Bisecting Glycans Prefer to Link the Sole GlcNAc Branch

Bisected GlcNAc is also an important modification to the *N*‐glycan core catalyzed by *N‐*acetylglucosaminyltransferase‐III (GnT‐III/ MGAT‐III).^[^ ^34^ ^]^ Among the top 10 down‐regulated site‐specific glycans, 7 contained bisected core structures (with/without core fucosylation) and 7 had at least one sole GlcNAc branch structure with 6 overlaps (Figure 4d). Similarly, among all down‐regulated glycopeptides in the middle‐aged thymus (FC > 1.5, *p* < 0.05), 41.8% contained bisecting glycans and 57.9% also had a branch structure with sole GlcNAc (Figure 4f,g). When the cutoff was switched to FC > 2 and *p* < 0.05, the proportion of glycopeptides containing bisecting glycans and the sole GlcNAc branch structure was further increased to 48.7% and 71.7%, respectively, indicating that these two sub‐structures were important structural features in decreased *N*‐glycopeptides during thymic involution (Figure S5d,e, Supporting Information). Interestingly, 81.4% of down‐regulated IGPs with bisecting *N*‐glycans were accompanied by at least a sole GlcNAc branch structure, while only 37.9% of up‐regulated glycopeptides with bisecting *N*‐glycans contained the sole GlcNAc branch structure (Figure 6i). Instead, these up‐regulated bisecting glycans mainly contained the LacNAc (61.9%), sialyl‐LacNAc (5.0% Neu5Ac and 3.2% Neu5Gc), and a small amount of Lewis^x/a^, sLewis^x/a^, and LacdiNAc branch structures (Figure 6j). These results suggested that the branch structures attached to the bisecting glycans may have become more complex and diverse during thymic involution. In addition, the up‐ and down‐regulated bisecting glycoproteins exhibited different biological functions. The glycoproteins with up‐regulated bisecting glycans were mainly involved in cell‐cell adhesion, tissue development, antigen processing, and presentation, and T‐cell mediated cytotoxicity; while glycoproteins with down‐regulated bisecting glycans were predominantly associated with positive regulation of immune system processes, innate immune response, and defense response to Gram‐positive bacterium (Figure 6k).

#### 2.5.4 Glycan Structure Variations at the Same Glycosites During Thymic Involution

During the gene ontology analyses, we found that many up‐ and down‐regulated glycopeptides were co‐enriched in the same GO categories, but displayed different proportions of core and branch structures. These co‐enriched GO categories included cell adhesion, immune system processes, and integrin‐mediated signaling pathways (Figure 4h). The up‐regulated glycopeptides displayed higher proportions of the Core‐I and sialylated branch structures, whereas down‐regulated glycopeptides possessed more Core‐III and the sole GlcNAc branch (Figure S8, Supporting Information). Further analyses showed that many up‐ and down‐regulated glycopeptides were derived from the same glycoproteins or even the same glycosites (Figure 6l). By comparing detailed glycan structures between up‐ and down‐regulated glycopeptides derived from either the same glycoproteins or the same glycosites, we confirmed our above observations that the up‐regulated glycopeptides displayed a higher prevalence of Core‐I and sialylated branch structures, whereas the down‐regulated glycans exhibited a higher prevalence of Core‐III and the sole GalNAc branch (Figure 6m–o). These results suggested that thymic involution was accompanied by an increase in sialylated glycan structures and a decrease in Core‐III and GlcNAc‐type glycosylation. Notably, these opposing trends were observed not only across different glycoproteins but also at the same glycosites, suggesting possibly site‐specific remodeling of glycan structures during thymic involution. (Figure 6o).

### 2.6 Regulatory Network Analysis Offers a Broader Perspective for Understanding Glycosylation Functions and Related Mechanisms

**Figure 7:**
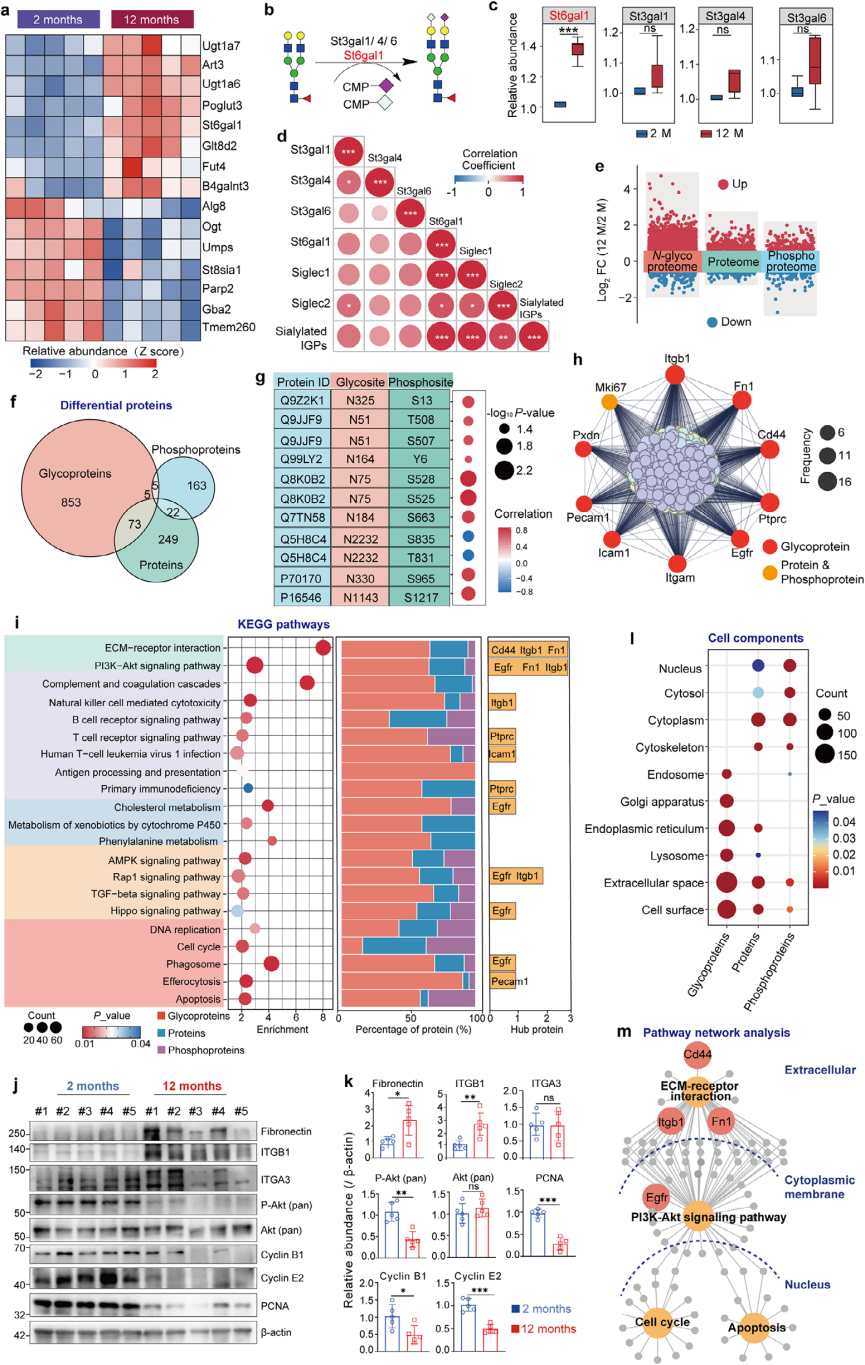
Integrated multi‐omic analyses to uncover insights into glycosylation function and the potential mechanism of thymic involution. a) Heat map showing significantly altered glycosyltransferases in mouse thymic involution. b) Schematic of sialoglycan synthesis pathways. c) The boxplots showing the expressions of four sialyltransferases based on the global proteome data. *n* = 5 per group. *p*‐values were calculated using unpaired *t*‐tests. ^***^ *p* < 0.001; ns, not significant. d) Spearman correlation analysis between the abundance of sialoglycans, sialyltransferases, and sialic acid‐binding proteins. e) Volcano maps of quantitative *N*‐glycoproteome, proteome, and phosphoproteome. f) Comparison of altered glycopeptides with altered phosphopeptides and proteins. The glycoprotein and phosphoprotein numbers indicate that the proteins had at least one changed glyco‐ and phospho‐peptide in the middle‐aged thymus. g) Correlation analysis between co‐existing glycosylation and phosphorylation sites on the same proteins. Spearman partial correlation coefficients were calculated between glycosylation and phosphorylation sites on the same protein, controlling for the effect of total protein abundance. Statistical significance was defined as *p* < 0.05. Correlation was considered notably positive if *r* > 0.05 and notably negative if *r* < −0.05. h) Top 10 hub proteins screened from all differential proteins from multi‐omic data based on the String and Cytoscape. Red indicates that the hub proteins were only altered at the glycopeptide level, while yellow indicates the hub proteins altered at both the phosphorylation and protein expression levels. i) KEGG pathways involved by differential glyco‐, phospho‐, and global proteins (left panel). Their percentages are shown in the middle panel, and the related top 10 hub proteins are shown in the right panel. j) Western blot analysis of key proteins involved in ECM–receptor interaction, PI3K–AKT signaling, and cell cycle regulation in mouse thymus. Representative Western blot images are present to show the expression levels of ITGB1 and fibronectin (ECM‐receptor interaction), phosphorylated AKT (PI3K‐AKT pathway), and Cyclin B1, Cyclin E2, and PCNA (cell cycle) in thymic tissues from 2‐month‐old to 12‐month‐old mice. For each age group, samples #1–3 represent female mice, and samples #4–5 represent male mice. k) Quantification of protein expression levels based on grayscale intensities in Figure 7k. Protein expression levels were normalized to β‐actin and compared between the two age groups. Data are presented as mean ± SD. Statistical significance was assessed using an unpaired two‐tailed *t*‐test. *p* < 0.05 was considered significant. l) Subcellular locations of differentially expressed glyco‐, phospho‐, and global proteins co‐enriched in the same KEGG pathways. m) Four pathways were interconnected and formed a signaling network. The glycoproteins altered at the glycosylation level were mainly located in the extracellular parts, whereas the differential phosphoproteins or proteins were mainly located in the intracellular parts. The Enrichment Network Plot was generated using OmicStudio tools (https://www.omicstudio.cn/tool).

We finally performed regulatory network analyses of altered glycans using multi‐omics data, since almost all biological processes are driven by multi‐level molecular cooperation within an integrated network system. We first investigated the involution‐related changes in glycosyltransferases, glycosidases, and glycan‐binding proteins using the quantitative proteome data. Among 164 glycosyltransferases identified in mouse thymus, 15 exhibited notable expression differences (*p* < 0.05) (**Figure** **7**a; Figure S9a, Supporting Information). Especially, among the four detected sialyltransferases responsible for sialylation, the α‐2,6 sialyltransferase St6gal1 was upregulated, whereas all three α2,3 sialyltransferases, including St3gal1, St3gal4, and St3gal6, showed no significant differences between the two age groups (Figure 7b,c). The upregulation of St6gal1 and stable expression of St3gal1, St3gal4, and St3gal6 were further validated by immunohistochemistry assays (Figure S9b), suggesting that the up‐regulated sialoglycopeptides might be caused by the upregulated St6gal1 in the middle‐aged thymus. Among two known glycosyltransferases (B4galnt3 and B4galnt4) responsible for the biosynthesis of LacdiNAc glycans, B4galnt3 was detected in the proteomic data and also showed an overexpression in the middle‐aged thymus. Our proteomic data also revealed 8 significantly changed glycosidases in the middle‐aged thymus (*p* < 0.05, Figure S9c,d). Interestingly, we observed significant alterations in the *O*‐GlcNAc glycosyltransferase OGT and the glycosidase OGA, even though the *O*‐GlcNAcylation was not the focus of this study.

We also measured alterations of the related glycan‐binding proteins based on the proteomic data, which might interact and cooperate with altered glycans. Among 147 glycan‐binding proteins identified in mouse thymus, 54 exhibited notable expression differences (*P* < 0.05). Notably, sialic acid‐binding proteins, including Siglec‐1 and Siglec‐2, were upregulated during thymic involution (Figure S9e–g, Supporting Information). Especially, a significant positive correlation was observed among the elevated sialylated IGPs, their sialyltransferase St6gal1, and the related glycan‐binding proteins Siglec‐1 and Siglec‐2, suggesting that these several types of biomolecules might interact or cooperate to perform essential functions during the thymic involution in mice (Figure 7d).

By integrating with phospho‐ and global proteomic data, we also investigated the signaling pathways that were possibly involved by the altered site‐specific glycans (Figure 7e,f). From 8837 quantified proteins and 6063 phosphopeptides (corresponding to 1993 phosphoproteins), we identified 265 upregulated and 84 downregulated proteins as well as 162 increased and 84 decreased phosphorylation sites (FC > 1.5, *p* < 0.05) (Figure S10a–f; Tables S4 and S5, Supporting Information). Although the crosstalk between phosphorylation and *O*‐GlcNAcylation on the same protein has been previously reported,^[^ ^35^, ^36^, ^37^ ^]^ few studies have focused on the crosstalk between *N*‐glycosylation and phosphorylation. Interestingly, we identified 49 proteins carrying both glycosylated and phosphorylated sites, among which 9 pairs showed strong positive correlations (*r* > 0.5) while the other 2 pairs exhibited negative correlations (*r* < −0.5) (Figure 7g), suggesting potential regulatory crosstalk between these two important modifications on the same proteins.

Based on the integrated analyses of all differential glyco‐, phospho‐, and global proteins, we found that nine of the top 10 hub proteins were actually glycoproteins, and all their alterations mainly occurred at the glycosylation level (Figure 7h). The signaling pathways involved by these altered proteins could be classified into five categories, including the ECM‐receptor interaction, immunity, metabolism, signaling, as well as cell proliferation and death (Figure 7i). Many of these pathways were further confirmed by using Western blotting, including the ECM‐receptor interaction pathway by detecting the upregulated ITGB1 and fibronectin, the PI3K‐Akt signaling pathway based on downregulated phosphorylated AKT, and the cell cycle through the related proteins such as decreased Cyclin B1, Cyclin E2, and PCNA in 12‐month‐old mice (Figure 7j,k). We also found that 85.7% of these KEGG pathways were comprised of at least 50% glycoproteins, and 52.3% of pathways contained at least one hub glycoprotein (Figure 7i). Since most glycoproteins enriched in above KEGG pathways were localized at the cell membrane, extracellular space, and cell surface, while proteins and phosphoproteins are primarily situated in the cytosol and nucleus (Figure 7l), it is unsurprising to find that four pathways including the ECM‐receptor interactions, PI3K‐Akt signaling pathway, apoptosis, and the cell cycle were interconnected and formed a signaling cascade from extracellular to intracellular transmission axes (Figure 7m). These data suggested that *N*‐glycans at the cell surface or matrix glycoproteins might play pivotal roles in initiating signal transduction during thymic involution.

## 3 Discussion

For a long period, large‐scale analyses of structural and site‐specific glycans have been known as one of the most challenging tasks in the proteomics and PTM omics fields.^[^ ^3^ ^]^ Fortunately, many site‐specific glycoproteomic software tools have been developed in recent years for large‐scale intact glycopeptide analyses.^[^ ^4^ ^]^ With these promising advances, how to extract all important glycosylation information from these multi‐dimensional glycoproteomic data has become another issue to be resolved. This is an important prerequisite before these omics approaches can be widely applied to various biomedical samples. In this study, we uniquely addressed this gap by developing a comprehensive data mining strategy to systematically extract overall and differential glycan features from high‐resolution quantitative *N*‐glycoproteomic data.

When applying the strategy to glycoproteomic analyses of mouse thymic involution, we first obtained an in‐depth and high‐resolution glycoproteome map of the mouse thymus. In addition to 18652 identified IGPs with 647 defined *N*‐glycan structures attached at 3503 glycosite‐containing peptides, we also identified a total of 5910 *N*‐glycosites and 3746 *N*‐glycoproteins from mouse thymus within 1% FDR at the glycosite‐containing peptide level. All these spectra contained the oxonium ions and Y1‐Y5 patterns for the core structures of *N*‐glycans, and therefore, these identified *N*‐glycosites have much higher confidence than most previously reported glycosites, only based on their de‐glycosylated forms.^[^ ^38^, ^39^ ^]^ Based on the overall profile of site‐specific glycans, besides the overall glycan isomers and sub‐structural features observed in the mouse thymus, we interestingly found that many cellular components exhibited distinct glycan structure patterns, especially much higher proportions of LacdiNAc and Lewis epitopes were detected in the MHC class I complex than other immune‐relevant cellular components. These interesting observations mainly benefited from the simultaneous determination of detailed glycan structures and their attached glycosites, so that all structural features of glycans could be linked to certain subcellular locations, biological processes, and signaling pathways via their attached glycoproteins. This is an important step toward further functional and mechanistic studies from a glycobiological perspective.

When extracting the altered glycan features from quantitative glycoproteomic data, we realized that each glycan or structural feature had different numbers of IGPs, and the numbers of up‐ and down‐regulated IGPs associated with thymic involution were also different. It was therefore difficult to extract all highly altered glycans or structural features only based on the numbers of the altered IGPs. We therefore used several different types of proportions to eliminate their effects to recognize the overall or uniquely up‐ and down‐regulated glycan features, as well as the glycan structural features changed at high proportions or high fold‐changes. In addition, we also used the normalized glycosylation to further eliminate the effects of their protein expression changes. Using these strategies, we observed four major glycan structure alterations with thymic involution. Especially, the elevated sialoglycopeptides and LacdiNAc glycans were further validated by the increased expressions of sialyltransferase St6gal1 and glycosyltransferases B4galnt3/4, respectively. These findings align with recent studies showing that glycosyltransferase expression influences glycan composition across tissues and diseases,^[^ ^40^, ^41^ ^]^ supporting a regulatory link between enzyme expression and sialylated or LacdiNAc glycan profiles during thymic involution.

Integrated multi‐omic analyses provided additional information to reveal multi‐level molecular cooperations. Taking the increased sialylation as an example. Integrated glyco‐, phospho‐, and global proteome analyses not only showed that the upregulated sialoglycopeptides mainly occurred at the glycosylation level, most likely through an α2,6‐linkage, but also revealed that they might interact with the overexpressed Siglec‐1 and Siglec‐2 to finally perform functions through several activated signaling pathways during thymic involution. In particular, four pathways, including the ECM‐receptor interaction, PI3K‐Akt signaling, cell cycle, and apoptosis pathways were found to be closely linked and formed a signaling cascade from extracellular to intracellular transmission axes. Given the fact that glycoproteins are predominantly localized at the cell surface or extracellular matrix, glycosylation might perform distinct roles in signal transduction compared to phosphorylated and global proteins that are primarily localized within the cytoplasm and nucleus.

## 4 Conclusion

In conclusion, we present a comprehensive data mining strategy coupled with our StrucGP software for systematic extraction of overall and differential glycan features from quantitative glycoproteomic data. By applying the strategy to the thymic involution studies, we show that the method can not only produce the most comprehensive glycoproteome map of the mouse thymus to date and screen out many distinct glycan structure patterns from different subcellular locations or biological processes but also effectively extract four major glycan structure alterations with age‐related thymic involution. Integrated multi‐omics analyses further confirm the cooperation of altered glycans with their upstream glycosyltransferases, glycosidases, glycan‐binding proteins, and downstream signaling pathways. Given its outstanding performance, the strategy should have broad application prospects in an in‐depth investigation of overall and altered glycan features from various biomedical samples.

## 5 Experimental Section

### Animals and Tissue Collection

The C57BL/6J mice (*n* = 44) were purchased from Jiangsu Huachuang Xinnuo Pharmaceutical Technology (Jiangsu, China) at young (2‐month‐old, 20.49 ± 3.5 g, 7 male and 15 female) and middle age (12‐month‐old, 33.67 ± 2.5 g, 7 male and 15 female). All mice were maintained under temperature‐controlled (20–25 °C) and humidity‐controlled (55 ± 5%) conditions with a 12 h light/dark cycle and free access to food and water. The mice were anesthetized with an intraperitoneal injection of pentobarbital sodium (0.6 mg g^−1^ body weight), and cardiac perfusion was performed to minimize blood interference in subsequent experiments. The thymus was dissected and washed twice with pre‐cooled PBS buffer. The harvested tissues were weighed. One female and one male mouse were randomly selected from each age group to obtain thymus tissue, which was fixed and paraffin‐embedded, while the remaining thymus tissues were collected from all mice, frozen in liquid nitrogen, and stored at ‐80 °C for further experiments. All procedures complied with ethical regulations and were approved by the Ethics Committee of Northwest University (Approval No. NWU‐AWC‐20240902M).

### Hematoxylin‐Eosin (H&E) Staining

The H&E staining was performed using an H&E staining kit (Cat. YK2222, Y&K Bio, China). Paraffin‐embedded thymus sections were cut into 5 µm sections. The sections were dewaxed and hydrated, and then stained with hematoxylin for 5 min. After a wash under running water, the sections were differentiated with 1% hydrochloric acid alcohol for several seconds. The sections were washed under running water, then treated with 0.6% ammonia water to return to blue, and washed with running water again. The sections were stained with eosin solution for 30 s, dehydrated, and sealed in neutral resin. The H&E‐stained sections were observed under an automatic digital slide scanning imaging system (Slidescan, China).

### Immunohistochemistry

Immunohistochemistry was performed as described previously.^[^ ^42^ ^]^ Briefly, pretreated thymus tissue sections were blocked and incubated with anti‐B4GALNT3 antibody (Cat. orb31745l, Biorbyt, 1:100), anti‐B4GALNT4 antibody (Cat. orb546266, Biorbyt, 1:1000), anti‐ST6GAL1 antibody (Cat. 14355‐1‐AP, Proteintech, 1:100), anti‐ST3GAL6 antibody (Cat. 13154‐1‐AP, Proteintech, 1:100), anti‐ST3GAL1 antibody (Cat. orb1294261, biobyt, 1:100), or anti‐ST3GAL4 antibody (Cat. 13546‐1‐AP, Proteintech, 1:100) overnight at 4 °C. The secondary antibody reagent was then added to completely cover the tissue, and incubated at room temperature for 20 min. Staining was visualized using a DAB chromogenic kit (Cat. DAB‐1031, MXB, China). The sections were observed under an automatic digital slide scanning imaging system (Slidescan, China), and 5 different fields of view were randomly selected in each section for quantification.

### Masson's Trichrome and Sirius Red Staining

The collagen content was assessed using Masson's Trichrome (Cat. YK2223, Y&K Bio, China) and Sirius Red Staining Kit (Cat. YK2224, Y&K Bio, China) according to the manufacturer's instructions. In brief, thymus paraffin tissue sections were deparaffinized with xylene and dehydrated in different concentrations of ethanol. The sections were stained with Masson's Trichrome and Sirius red. Collagen fibers appeared red in Sirius Red staining and blue in Masson staining. The sections were observed under an automatic digital slide‐scanning imaging system (Slidescan, China) and quantified using Image J software (v.1.52).

### Periodic Acid‐Schiff Staining

Glycogen was performed with a Periodic Acid‐Schiff (PAS) assay kit (Cat. YK2206, Y&K Bio, China) according to the manufacturer's instructions. Periodic acid oxidation converted intracellular polysaccharides to a dialdehyde, which reacted with Schiff's reagent to produce a red coloration. The sections were observed under an automatic digital slide scanning imaging system (Slidescan, China) and quantified using the Image J software (v.1.52).

### Fluorescence‐Based Lectin Histochemistry

Fluorescence‐based lectin histochemistry was conducted to analyze LacdiNAc structures on MHC molecules in mouse thymic tissues, following a previously described protocol with minor modifications.^[^ ^43^ ^]^ Formalin‐fixed paraffin‐embedded (FFPE) tissue sections were first deparaffinized in xylene and rehydrated through a graded ethanol series. After rehydration, sections were rinsed in phosphate‐buffered saline (PBS) and blocked for 1 h at room temperature (RT) with 5% (w/v) bovine serum albumin (BSA) containing 0.04% Triton X‐100. The sections were then incubated overnight at 4 °C in the dark with either anti‐MHC class I antibody (sc‐59199, Santa Cruz, 1:50) or anti‐MHC class II antibody (sc‐59322, Santa Cruz, 1:50), together with Cy5‐conjugated Wisteria floribunda agglutinin (WFA, L‐1350, Vector Laboratories) to detect LacdiNAc. After primary incubation, FITC‐conjugated secondary antibodies (PMK‐014‐094 M, Biopmk, 1:1000) were applied for 1 h at RT in the dark. Nuclear counterstaining was performed using DAPI (C1005, Beyotime) for 10 min. Fluorescence imaging was performed using a Leica TCS SP8 confocal microscope (Leica Microsystems, Wetzlar, Germany). Quantitative analysis of fluorescence signals was carried out using ImageJ software (version 1.52).

### Western Blot Analysis

Mouse thymus tissues were freshly collected, weighed, and homogenized in RIPA lysis buffer supplemented with Protease inhibitor cocktail (HY‐K0010, MCE) and phosphatase inhibitors (P1081, Beyotime) using an automatic tissue homogenizer (Jing Xin, China) at low temperature. Homogenates were incubated on ice for 30 min and centrifuged at 12 000 × g for 10 min at 4 °C. Supernatants were collected, and protein concentrations were measured using a BCA Protein Assay Kit (P0012, Beyotime).

Equal amounts of protein were separated by SDS‐PAGE and transferred to PVDF membranes (Millipore, USA). After blocking with 5% non‐fat milk, membranes were incubated overnight at 4 °C with the following primary antibodies (1:1000 dilution): phospho‐AKT1/2/3 (Ser472/473/474, F1644, Selleck), pan‐AKT (F0004, Selleck), PCNA (F0018, Affinity), Fibronectin (CY5621, Abways), Cyclin E2 (4132T, CST), Cyclin B1 (28603‐1, Proteintech), ITGB1 (ab30394, Abcam), ITGA3 (ab131055, Abcam), HRP‐conjugated β‐actin (HRP‐60008, Proteintech), and HRP‐conjugated GAPDH (HRP‐60004, Proteintech). Following washing, membranes were incubated with HRP‐conjugated secondary antibodies (1:5000, 1 h, RT): goat anti‐rabbit IgG (RGAR001, Proteintech) or goat anti‐mouse IgG (RGAM001, Proteintech). Signals were detected using ECL reagents (WBKLS0050, Millipore, USA) and visualized with a chemiluminescence imaging system (Qinxiang, China).

### Protein Extraction and Digestion

Thymus tissues were cut into ≈1 mm^2^ fragments and washed three times with precooled phosphate‐buffered saline (PBS, pH 7.4). Subsequently, the thymus tissues were denatured in an 8 m Urea (Cat. U5128, Sigma, Germany)/ 1 m NH_4_HCO_3_ (Cat. 11213, Sigma, Germany) lysis solution, homogenized through physical grinding with precooled steel balls using an automatic rapid tissue homogenizer (Shanghai Jing Xin, China) at 60 Hz for 2 min, and sonicated until the solutions became clear. Lysates were centrifuged at 15 000 g for 10 min at room temperature, and the supernatants were collected for protein BCA quantification (Cat. P0012, Beyotime, China). Due to the limited tissue size, thymus proteins from 3 to 5 mice were pooled together to form five biological replicates (two males and three females) per age group. Proteins were reduced with 5 mm dithiothreitol DTT solution (Cat. 43819, Sigma, Germany) at 37 °C for 1 h. The proteins were then alkylated with 15 mm iodoacetamide (IAM) (Cat. I6125, Sigma, Germany) for 30 min at room temperature (RT) in the dark, followed by the addition of 2.5 mm DTT for 10 min at RT. The protein solutions were diluted twofold with HPLC‐grade water for the first cycle of digestion, 100:1 sequencing grade trypsin (Cat. V5113, Promega, USA) was added, and enzymatic hydrolysis was performed at 160 r min^−1^ and 37 °C for 2 h. After the initial digestion, the solution was further diluted fourfold with HPLC‐grade water, 100:1 sequencing grade trypsin was added, and shaken at 160 r min^−1^ and 37 °C overnight. The enzymatically hydrolyzed solutions were acidified with trifluoroacetic acid (TFA) (Cat. 3020031, Sigma, Germany) to reach pH < 2 and centrifuged at 15,000 g to remove particulate matter. Peptides were desalted using an Oasis C18 column (Cat. WAT054955, Waters, USA) and eluted in a 60% ACN (A9554, Thermo Fisher Scientific, USA) / 0.1% TFA solution. The peptide concentrations were determined by measuring UV absorbance at 215 nm using a DS‐11 spectrophotometer (Denovix, Wilmington, DE, USA).

### Tandem Mass Tag (TMT) Labeling

Equal amounts of tryptic peptides from each pooled sample (five young and five middle‐aged) were labeled with one channel of 10‐plex TMT reagents following the manufacturer's protocols (Cat. YA332834, Thermo Fisher Scientific, USA). The TMT channels were listed as follows: five young pooled samples were labeled by the channels 126, 127N, 127C (3 females), 128N, and 128C (2 males). While five middle‐aged pooled samples were labeled by the channels of 129N, 129C, 130N (3 females), 130C, and 131 (2 males). Before labeling, the pooled samples from each group were reconstituted in 50 mm HEPES (pH 8.5) (Cat. H3375, Sigma, Germany). The peptides were incubated with TMT reagents overnight at RT. Subsequently, 5% hydroxylamine (Cat. L13168, Thermo Fisher Scientific, USA) was added to terminate the reaction. Finally, all ten TMT‐labeled samples were then combined into a single pooled sample and desalted using a C18 column (Cat. WAT054955, Waters, USA). Approximately 5% of the pooled sample was used directly for global proteomic analysis via 2D‐LC‐MS/MS, while the remaining 95% was used for sequential enrichment of glyco‐ and phospho‐peptides for glycoproteomic and phosphoproteomic analyses.

### Intact Glycopeptide Enrichment

Intact glycopeptides were enriched from TMT‐labeled peptide samples using an Oasis Mixed Anion‐Exchange (MAX) column (Cat. 186000366, Waters, USA).^[^ ^9^ ^]^ Peptides eluted from the C18 column in 60% ACN/ 0.1% TFA were diluted to a final concentration of 95%ACN/ 1%TFA with 100% ACN and 10% TFA solution. Before application, the MAX column underwent three washes with 100% ACN, 100 mm triethylammonium acetate (Cat. 69372, Sigma, USA), HPLC grade water, and finally with 95% ACN/ 1% TFA. After sample loading, the MAX column was washed three times with 95% ACN/ 1% TFA to ensure proper enrichment. The glycopeptides bound to the MAX column were ultimately eluted twice with 0.2 mL of 50% ACN/ 0.1% TFA solution.

### Phosphopeptide Enrichment

Phosphopeptides were enriched using immobilized metal affinity chromatography (IMAC) as previously described.^[^ ^44^ ^]^ Briefly, the Fe^3+^‐NTA agarose beads were freshly prepared using Ni^2+^‐NTA super flow agarose beads (Cat. 30210, Qiagen, USA) and resuspended in ACN/ Methanol/ 0.01% acetic acid solution with volume ratio of 1:1:1. The glycopeptide‐enriched flow‐through peptide samples were adjusted to a solution comprising 80% ACN and 0.1% TFA, which was used for the enrichment of phosphorylated peptides. Peptide samples for IMAC were incubated with 80 µL of 25% (v/v) Fe^3+^‐NTA agarose beads to conjugate for 30 min with shaking at RT. The nonbinding peptides in the supernatant were collected by centrifugation. The beads should be resuspended using 80% ACN/ 0.1% TFA. Furthermore, the C18 Tip (Cat. 66883‐U, Supelco, Germany) was prepared for desalting phosphorylated peptides. The beads were put on the C18 Tip and were washed with 200 µL of bind/wash buffer by centrifugation. The peptides conjugated to beads were eluted twice with 80 µL 50% ACN/ 0.1% formic acid (FA) (Cat. A11750, Thermo Fisher Scientific, USA) solution. Enriched phosphopeptides were dried by vacuum concentration and resuspended in 20 µL of 3% ACN/ 0.1% FA solution for LC‐MS/MS analysis.

### High‐Performance Liquid Chromatography (HPLC) Fractionation

HPLC manipulations have been described in the previous studies.^[^ ^45^ ^]^ Briefly, dried IGPs, phosphopeptides, and peptides were resuspended in 2% ACN/ 20 mm NH_4_COOH and subjected to graded HPLC in an Agilent 1260 using an Agilent RP Zorbax 300 Å Extend C18 column (250 mm × 4.6 mm, OD 5 µm) via high pH reversed‐phase HPLC. The samples were separated into 96 fractions by a gradient of mobile phases A (2% ACN/ 5 mm NH_4_COOH, pH 10) and B (90% ACN/ 5 mm NH_4_COOH, pH 10) at a flow rate of 0.2 mL min^−1^. Gradient elution was performed for a total of 120 min (0–2% B for 10 min, 2–8% B for 5 min, 8–35% B for 85 min, 35–95% B for 5 min, and 95 to 95% B for 15 min). Peptides were detected at 215 nm and were collected along with the LC separation in a time‐based mode from 3 to 112 min. The 96 fractions of each sample were combined into 24 (glyco‐ and global peptides) or 12 fractions (phosphopeptides). The samples were then dried in a speed vacuum and stored at −80 °C until LC‐MS/MS analysis.

### LC‐MS/MS Analysis

The detailed LC‐MS/MS method has been described previously.^[^ ^46^ ^]^ About 1 µg labeled intact glycopeptides, phosphopeptides, and peptides were separated by an Easy‐nLC 1200 system (Thermo Fisher Scientific, San Jose, CA, USA) with the use of a 75 µm × 50 cm Acclaim PepMap‐100 C18 analytical column (Cat. 164570, Thermo Fisher Scientific, USA) protected by a 75 µm × 2 cm trapping column (Cat. 164946, Thermo Fisher Scientific, USA). The flow rate was kept at 300 nL min^−1^ with the mobile phase consisting of 0.1% FA in water A) and 0.1% FA in 80% ACN B), and the analytical column was heated and maintained at 50 °C. The samples were then analyzed by LC‐MS/MS on an Orbitrap Fusion Lumos Mass Spectrometer (Thermo Fisher Scientific, Germany). The gradient profile (130 min) for proteome and phosphoproteome was set as follows: 3–10% B for 3 min, 10–30% B for 92 min, 30–68% B for 8 min, 68–98% B for 3 min, equilibrated in 2% B for 1 min and 98% B for 5 min for 2 cycles, and 2% B for 12 min. The spray voltage was set at 2.4 kV, MS1 spectra (AGC 4.0 × 10^5^) were collected from 350–1800 m/z at a resolution of 60 K followed by data‐dependent HCD tandem mass spectrometry (HCD‐MS/MS) with the collision energy of 36%, RF lens at 30%, and intensity threshold of 5.0 × 10^4^. The charge states for peptides were 2–5. A dynamic exclusion time of 20 s was used to discriminate against previously selected ions for MS/MS fragmentation.

For glycoproteomics, a 120 min gradient was applied as follows: 3–7% B for 3 min, 7–35% B for 80 min, 35–68% B for 8 min, 68–99% B for 4 min, 99–2% B for 1 min, 2–99% B for 5 min, 99–2% B for 1 min, and 2–99% B for 5 min, followed by 2% B for 13 min. The spray voltage was set at 2.4 kV. Orbitrap MS1 spectra (AGC 4.0 × 10^5^) were collected from 375–1800 m/z at a resolution of 50 K. Selected precursor ions (charge states of 2–5) were isolated with a m/z 2 width and subjected to data‐dependent HCD fragmentation at 50K resolution. Two MS2 spectra were generated per precursor, one using low‐energy HCD (27%) for glycan structure analysis and another using high‐energy HCD (40%) for peptide sequence identification. Dynamic exclusion was set to 20 s to minimize the re‐selection of previously fragmented ions.

### Database Search for Proteome and Phosphoproteome

All LC‐MS/MS data were searched against the reviewed *Mus musculus* reference proteome database (UniProt, Proteome ID: UP000000589, downloaded on January 11, 2024). Peptides and phosphopeptides were identified by Thermo Proteome Discoverer 3.1 (Thermo Fisher Scientific, Germany). The search parameters were set as follows: up to two missed cleavage sites were permitted for trypsin digestion, and the mass tolerances of precursors and fragment ions were set as 10 ppm and 0.02 Da, respectively. Carbamidomethylation (C, +57.0215 Da) and TMT10plex (N‐termini of peptides, +229.162 932 Da) were set as fixed modifications, and oxidization (M, +15.9949 Da), 10‐plex TMT (K, +229.162 932 Da), and deamidation (N, +0.98 Da) were set as dynamic modifications. The peptides were quantified based on intensities of TMT reporter ions (normalized by total intensities within the TMT set) in their identified MS/MS spectra. The phosphorylation (S/T/Y, + 79.966 Da) was also added as a variable modification for phosphoproteomic data. All results were filtered with a 1% false discovery rate (FDR). For the quantification of peptides and phosphopeptides, the intensities of TMT reporter ions were normalized within each TMT set to account for differences in sample loading and systematic variations across channels. Normalization was performed by dividing each TMT channel by the total sum of intensities across all channels within a set.

### Intact Glycopeptide Identification and Quantification

Intact glycopeptides were identified using the StrucGP software, which enables de novo glycan structure identification at the site‐specific level.^[^ ^9^ ^]^ As previously described,^[^ ^42^ ^]^ IGPs analyses were performed by StrucGP using the built‐in glycan branch structure database (17 branches) from StrucGP and the *Mus musculus* UniProt protein database. The protein enzymatic digestion was set as trypsin with a maximum of two missed cleavage sites, and the potential glycosite‐containing peptides were screened with the N‐X‐S/T motif (X is any amino acid except Proline). The carbamidomethylation (C, +57.0215 Da) was set as a fixed modification, and oxidization (M, +15.9949 Da) as a dynamic modification. The mass tolerances for MS1 and MS2 were set at 10 and 20 ppm, respectively. The identification results were filtered with 1% False discovery rate (FDR) for both peptide sequences and glycan structures, which was estimated by the decoy peptide method and decoy spectra method,^[^ ^6^, ^9^ ^]^ respectively. The search results were ranked based on their scores, and the peptide/glycan with the highest score was considered to be the corrected identification. In addition to FDR controls at both peptide and glycan levels, a probability strategy was used to further evaluate the reliability of each module of identified glycan structures.

For quantification, TMT reporter ions intensities were extracted from the identified MS/MS spectra with high‐energy HCD fragmentation (HCD = 40%) for relative quantification of intact glycopeptides. The intensities of TMT reporter ions were first normalized using global scaling factors obtained from global proteomic results to ensure consistency across the ten channels. These normalized intensities were used for quantitative analysis of the identified glycopeptide. The median intensities of all PSMs were calculated for each sample and were used to calculate the ratios of intact glycopeptides between the two age samples. To evaluate differences in glycopeptide abundances between two age groups, normalized data were statistically compared using two‐tailed unpaired t‐tests. Glycopeptides with a fold change greater than 1.5 or less than 0.67 and a *p*‐value < 0.05 were considered as differentially expressed IGPs.

### Bioinformatic Analysis

The Gene Ontology (GO) and Kyoto Encyclopedia of Genes and Genomes (KEGG) pathway analysis were performed using David (https://david.ncifcrf.gov/) and Metascape (https://metascape.org/). Go enrichment bubble plot was generated by GO. Figure (gitlab.com/evogenlab/GO‐Figure). Principal Component Analysis (PCA) and correlation analysis were performed using the OmicStudio tools (https://www.omicstudio.cn/) and GraphPad Prism 8.0.1. One‐way ANOVA was used to analyze the effect of age and gender on intact glycopeptides. The effect of gender is initially excluded, and the differences between 2‐month‐old and 12‐month‐old mice were assessed separately. The combined results of the one‐way ANOVA *p*‐value and the ANOVA effect size (η2) were employed to ascertain the impact of age and gender on the thymic involution *N*‐glycoproteome. Protein‐protein interaction (PPI) network analysis was performed using Search Tool for the Retrieval of Interacting Genes/ Proteins (STRING: functional protein association networks (string‐db.org)),^[^ ^47^ ^]^ and interactions with a combined score > 0.4 were selected to construct the PPI networks using Cytoscape.^[^ ^48^ ^]^ Furthermore, the CytoHubba and CytoNCA plugin was used to identify the hub proteins using 20 topological algorithms. Data visualization was completed by the bioinformatics online platform (http://www.bioinformatics.com.cn/), OmicStudio tools, and GraphPad Prism 8.0.1.

### Statistical Analyses

Data in the bar plots are shown as the mean ± SD. *p* <0.05 was considered to be statistically significant, ns indicates not significant. All experimental data were analyzed using unpaired *t*‐tests or Mann‐Whitney tests to compare differences between groups (GraphPad Prism 8.0.1)

## Conflict of Interest

The authors declare no conflict of interest.

## Author Contributions

Z.Z. and S.S. designed the experiments; Z.Z. performed sample preparation and MS analysis with help from Y.W., L.C., Y.C., and J.Z.; Z.Z. analyzed data with the help of K.H., Y.Z., Y.X., Y.Z., M.Y., and Z.J.; Z.Z. and S.S. wrote and edited the manuscript with help from all the authors.

## Supporting information



Supporting Information

Supplementary Table 1

Supplementary Table 2

Supplementary Table 3

Supplementary Table 4

Supplementary Table 5

## Data Availability

The mass spectrometry data have been deposited to the ProteomeXchange Consortium^[^ ^49^ ^]^ (http://proteomecentral.proteomexchange.org) via the iProX partner repository^[^ ^50^ ^]^ with the dataset identifier PXD058066 and PXD060000. The data that support the findings of this study are available from the corresponding author upon reasonable request.
