# Supplementary material for: A Glycoproteome Data Mining Strategy for Characterizing Structural Features of Altered Glycans with Thymic Involution
Source: Adv Sci (Weinh). 2025 Jul 24;12(38):e02013. doi: 10.1002/advs.202502013 (PMC12520470; doi:10.1002/advs.202502013)
Supplement: Supplementary file 1 — Supporting Information [file ADVS-12-e02013-s005.docx]

**Supporting Information for:**

**A Glycoproteome Data Mining Strategy for Characterizing Structural Features of Altered Glycans with Thymic Involution**

*Zhida Zhang, Yongqi Wu, Ke Hou, Yiwen Zhang, Lin Chen, Muyao Yang, Zhehui Jin, Yongchao Xu, Yingjie Zhang, Yinli Cai, Jiayu Zhao, Shisheng Sun^*^*

Laboratory for Disease Glycoproteomics, College of Life Sciences, Northwest University, Xi’an, 710069, P. R. China

*Corresponding author: [suns@nwu.edu.cn](mailto:suns@nwu.edu.cn)

**Supporting information**

1. **Supplementary Note**

Workflow of the Glycoproteome Data Mining Strategy.

1. **Supplementary Figures**

**Figure S1** Overall characterization of age-related thymic changes in mice.

**Figure S2** Large-scale profiling of intact glycopeptides in mouse thymus tissue.

**Figure S3** Identification of five different glycan isomers at a single N-linked glycosite.

**Figure S4** MS/MS spectra of identified hexa-antennary *N*-glycans.

**Figure S5** In-depth mining of glycosylation alterations during thymic involution.

**Figure S6.** Gene Ontology analysis of proteins carrying increased LacdiNAc glycans in middle-aged mouse thymus.

**Figure S7** Sialoglycans and *O*-acetylated sialoglycopeptides (*O*-AcSGPs) were largely up-regulated during mouse thymic involution.

**Figure S8** Analyses of glycan sub-structures in biological processes shared by up- and down-regulated glycopeptides.

**Figure S9** Dysregulation of glycosyltransferases, glycosidases, and glycan-binding proteins during mouse thymic involution.

**Figure S10** Quantitative proteomic and phosphoproteomic analyses of the mouse thymic involution.

1. **Supplementary Tables** (separate Excel files)

**Table S1** Intact glycopeptides identified in mouse thymus with FDR<1% applied at the glycosite-containing peptide level or both glycosite-containing peptide and glycan levels.

**Table S2** Summary of global proteins, glycoproteins, and phosphoproteins identified in mouse thymus.

**Table S3** Quantitative glycopeptides between young and middle-aged mouse thymus based on the TMT-labeling quantification.

**Table S4** Quantification of proteins between young and middle-aged mouse thymus based on the TMT-labeling quantification.

**Table S5** Quantification of phosphopeptides between young and middle-aged mouse thymus based on the TMT-labeling quantification.

**1. Supplementary Note**

**Workflow of the Glycoproteome Data Mining Strategy**

**Overview**

This workflow systematically summarizes and standardizes in-depth mining of site-specific glycan structural features in quantitatively structurala and site-specific glycoproteomics. It is particularly suitable for comprehensively characterizing the landscape and dynamic alterations of site-specific glycans under different physiological or pathological conditions.

**Section I: Global Characterization of Overall Site-Specific Glycan Structures**

**Step 1. Overview of Glycopeptide Identification (related to Figure 2, 3 and Figure S2)**
Identification depth of intact glycopeptides is assessed under two FDR control strategies:
- Within 1% FDR at the glycosite-containing peptide level;
- Within 1% FDR simultaneously at the peptide sequence and glycan structure level.
The following information can be obtained from the identification results of StrucGP: PSMs, unique intact glycopeptides (IGPs), unique peptides, glycosylation sites, glycoproteins, and N-X-S/T motif distribution.

**Step 2. Overview of Glycan Compositions and Structures (Figure 3 and Figure S2, 3)**

Sort glycan structures based on their compositions (HexNAc, Hex, Fuc, Neu5Ac/Neu5Gc) and count the structural isomers per composition. Present the top 10 glycan structures based on their modified glycosites, and analyze different glycan isomers attached at the same site.

**Step 3. Modular Structures Analyses of *N*-Glycans (Figure 3 and Figure S4, 7)**
Categorize glycan substructures by glycan type (high-mannose, hybrid, complex), core structure (Core I–IV), branch structures (LacNAc, LacdiNAc, Lewis^x/a^, single GlcNAc, etc), branch number, and special substructures (such as core fucose, sialylation, *O*-acetylation). Count the glycopeptides, glycosites or glycoproteins modified by each substructure, and calculate their proportions among all identified or specific types of glycopeptides

**Step 4. Glycan Substructures Patterns Across Compartments and Functions (Figure 3 and Figure S2)**
Integrates subcellular localization and GO enrichment to highlight differences across compartments and processes, and identify immune-relevant structural enrichments (e.g., MHC I, CD4/CD8).

**Section II: In-Depth Mining of Differential Site-Specific Glycan Features**

**Step 5. Normalization of Glycopeptide Quantitation**

Correct or normalize TMT channels using proteomics-derived factors and then the median ratio of each individual glycopeptide.

**Step 6. Selecting Differential Glycopeptides (Figure 4 and Figure S2)**
Select the differentially expressed IGPs using the threshold of FC > 1.5 and P < 0.05, or other similar cutoffs.

**Step 7. Top 10 Differential Glycan Structures (Figure 4)**
Rank top 10 up/down-regulated glycan structures based on thei modified glycosite counts.

**Step 8. Substructural Features of Differential Glycopeptides (Figure 4 and Figure S5)**

A systematic analysis of glycan substructural features was performed for differential glycopeptides, covering multiple dimensions including glycan subtypes, core structures (bisecting GlcNAc), branch structures (LacdiNAc, sialyl-LacNAc and sole GlcNAc branches), and branch counts. The relative proportions of each glycan substructure were calculated separately in upregulated and downregulated glycopeptides to identify substructure features specific to each regulation group.

**Step 9. Identification of Highly Variable Glycan Substructures (Figure 4 and Figure S5)**
Identify glycan substructures with the greatest number of differential occurrences, potentially serving as biomarkers.

**Step 10. Multi-Threshold Sensitivity Analysis (Figure S5, 9)**
Multi-threshold sensitivity analysis was conducted to investigate the variation trends of glycan substructures under different fold change cutoffs (e.g., FC > 1.2, 1.5, 2, 2.5, 3).

**Step 11. Eliminating Protein Abundance Effects(Figure S5)**Integrated quantitative proteomics analysis to determine whether differential glycopeptide changes arise from glycosylation alterations or protein abundance variations.

**Step 12. Glycan Remodeling at Shared Glycosites (Figure 6)**
Upregulated and downregulated glycopeptides derived from the same glycosylation sites are extracted. Structural remodeling of attached glycans are compared, focusing on patterns such as Core-III to Core-I conversion and shifts toward sialyl-LacNAc structures.

**Step 13. Focusing on Specific Glycopeptides (Figure 4, 5, 6, 7 and Figure S6, 7, 8, 11)**
Target glycopeptides exhibiting large fold changes, localized in key subcellular compartments (e.g., MHC I), or playing central roles in signaling pathways. These candidates are recommended for further functional validation, targeted drug development, or biomarker studies.


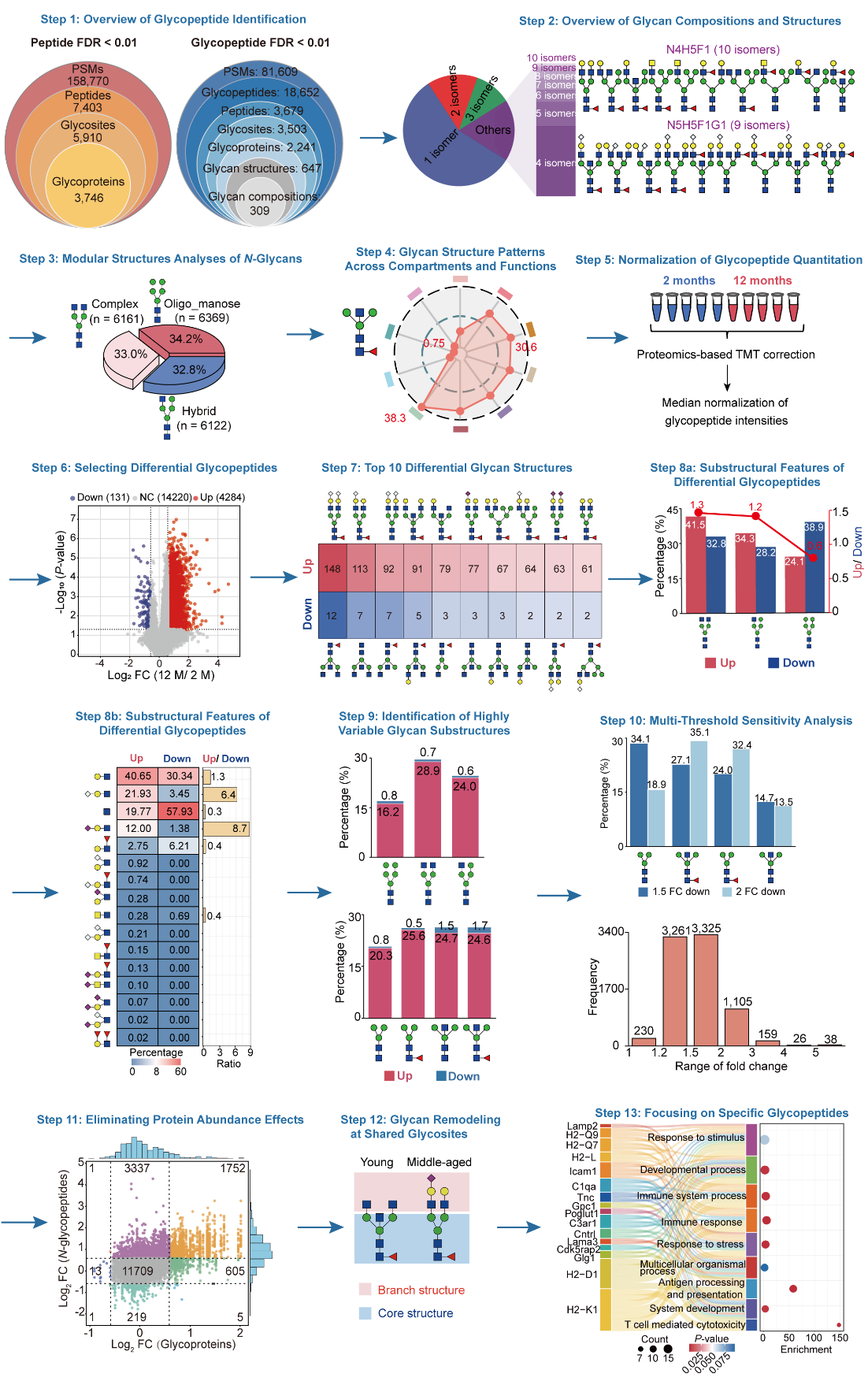


**Overview of the glycoproteomic data mining workflow**

This figure outlines the major steps of the glycoproteome data mining strategy, which integrates StrucGP-based identification of structural and site-specific glycans with a systematic pipeline for systematically extracting the overall and altered glycan structural features.

**2. Supplementary Figures**


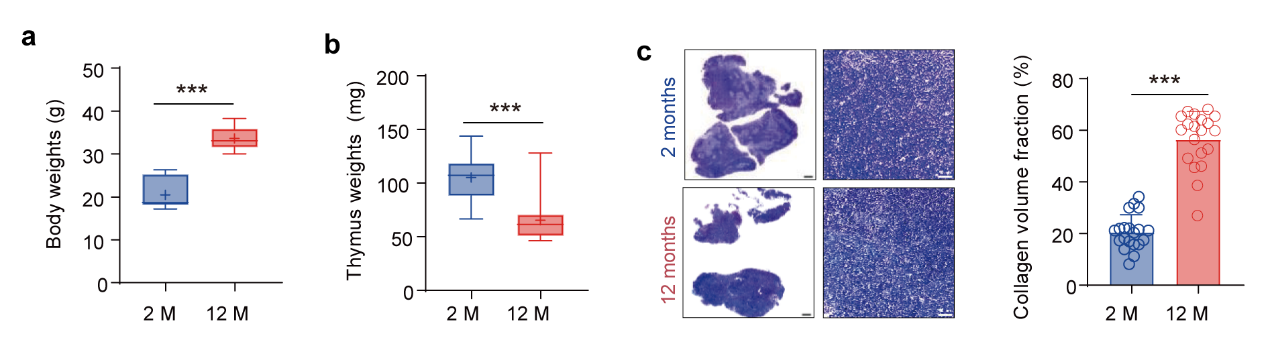


**Figure S1. Overall characterization of age-related thymic changes in mice. a,** Body weight of mice at 2 months (n = 20, 20.49 ± 3.5 g) and 12 months (n = 20, 33.67 ± 2.5 g) of age. **b,** Thymus weights of mice of both age groups. **c,** Collagen deposition was determined with Masson staining. Blue indicates collagen deposition (left panel, Scale bar, 500 μm), statistical analyses of Masson staining are shown in the right panel (n = 20). Related to Figure 2.

**
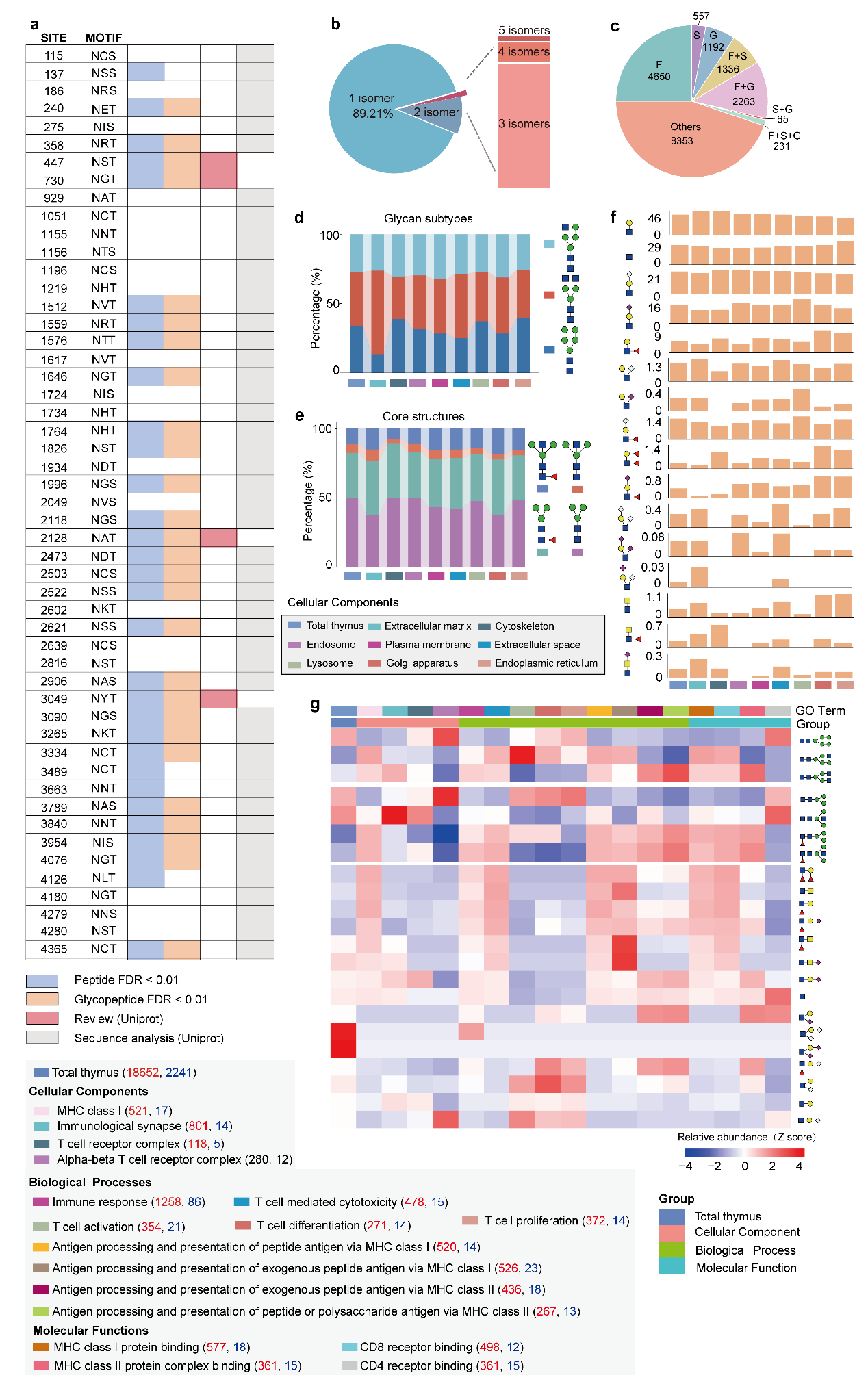
**

**Figure S2. Large-scale profiling of intact glycopeptides in mouse thymus tissue.** **a,** The glycoprotein with the highest number of N-linked glycosites (LRP1) that was identified in mouse thymus and these identified *N*-glycosites were compared to the UniProt database. **b,** Proportions of different glycan isomers identified at each glycosite. **c,** Numbers of fucose (F) and sialic acid (S/ G) on intact glycopeptides in mouse thymus tissue. Fucose (F), Neu5Ac (S), Neu5Gc (G). **d-f,** Proportions of different glycan subtypes (**d**), core structures (**e**), and branch structures (**f**) on glycoproteins that were located at different subcellular components in the mouse thymus. **g,** Comparison of different glycan sub-structures on glycoproteins that were involved in different immune-related gene ontology terms, including the cellular components, the biological processes, and the molecular functions. Related to Figure 3.

**
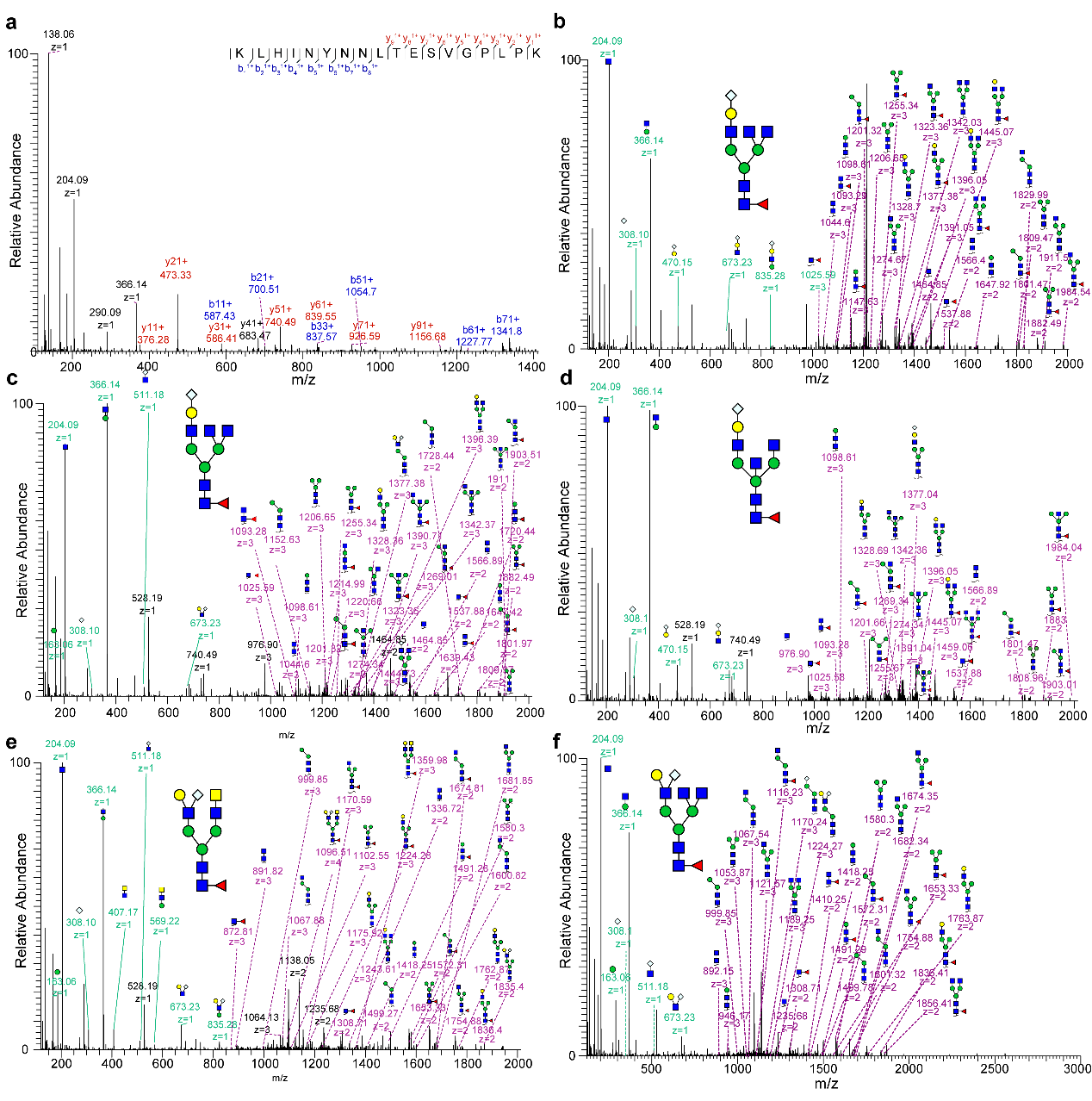
**

**Figure S3. Identification of five different glycan isomers from a single N-linked glycosite. a-f,** Representative MS/MS spectra of five isomers at a glycosite: peptide KLHINYNN#LTESVGPLPK modified by five glycans with the same composition N5H4F1G1 from lumican (LUM). The # indicates the N-linked glycosite. The sequence of the peptide was determined by matching the b and y ions (labeled in blue and red, respectively) in the high HCD energy (HCD = 40%, **a**) MS/MS spectra. The glycan structures were determined from the B and Y ions (labeled in green and purple, respectively) in the low HCD energy (HCD = 27%, **b-f**) MS/MS spectra. Related to Figure 3.

**
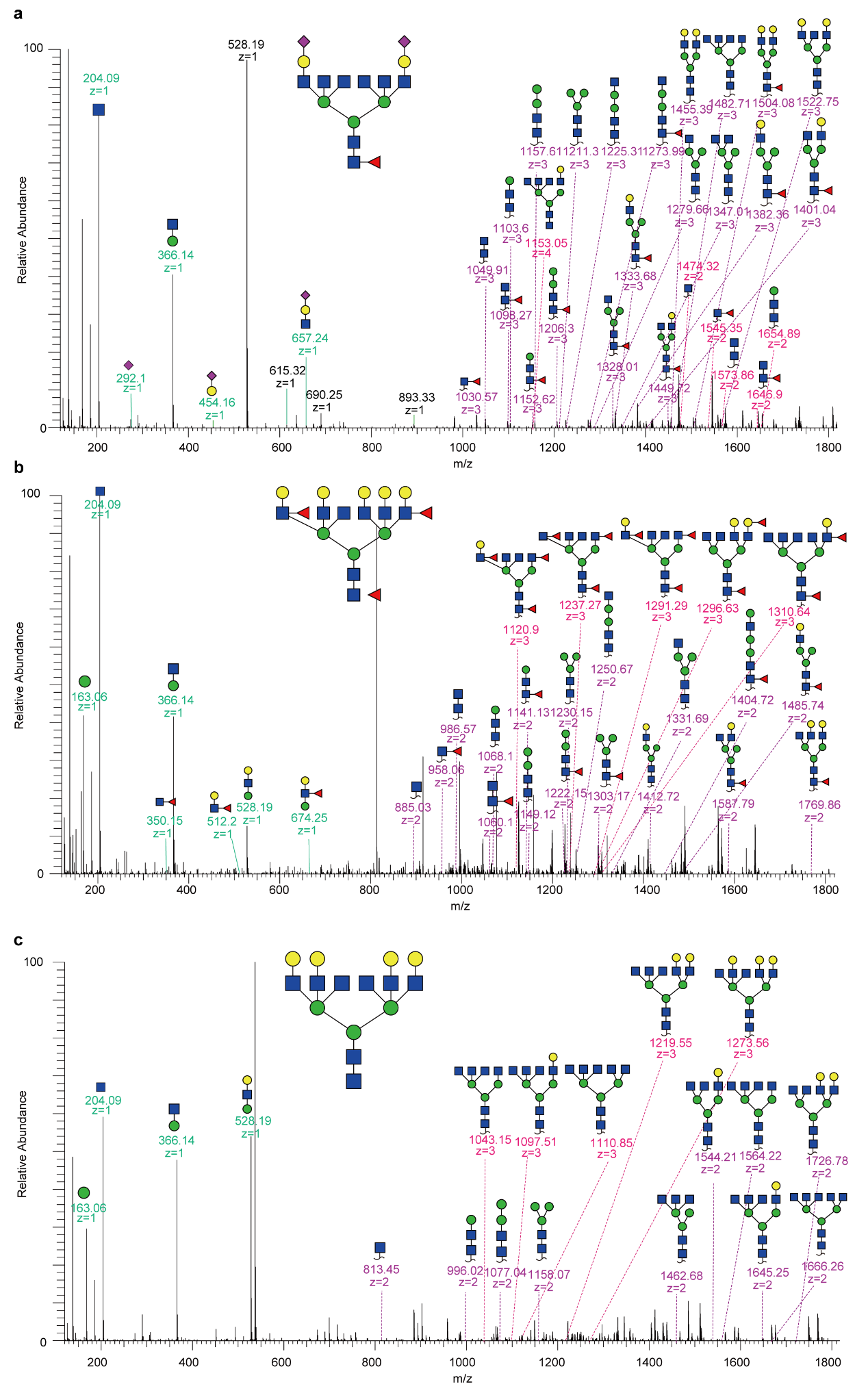
**

**Figure S4. MS/MS spectral of identified hexa-antennary *N*-glycans at the intact glycopeptide level.**

**
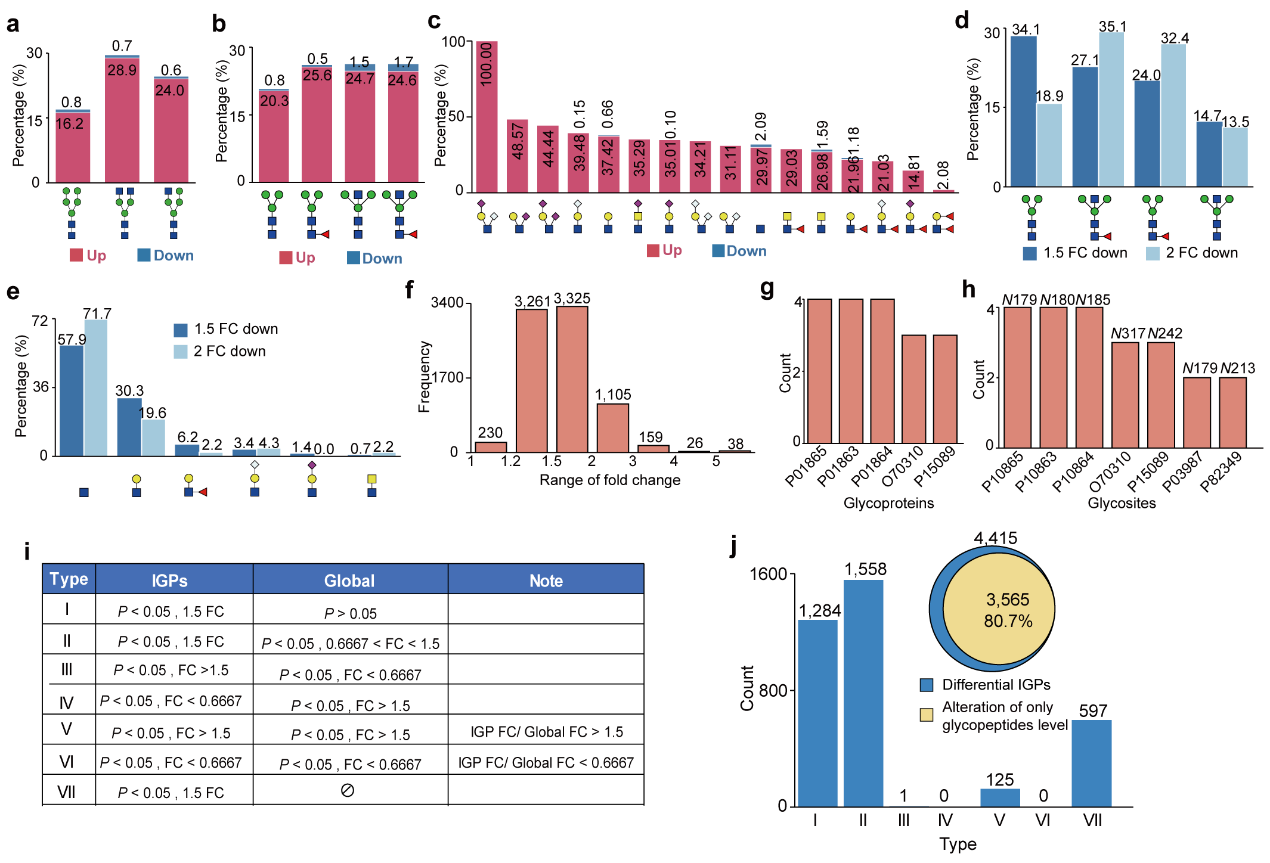
**

**Figure S5. In-depth mining of glycosylation alterations during thymic involution. a-c,** Comparing the proportions of glycan subtypes (**a**), core structures (**b**), and branch structures (**c**) between up- and down-regulated glycopeptides in middle-aged thymus. The proportion was calculated by dividing the numbers of up- or down-regulated glycopeptides with each sub-structure by the total numbers of all identified glycopeptides. **d, e,** The proportion of each core (**d**) and branch (**e**) structure among all down-regulated glycopeptides with at least a 1.5- or 2-fold change in the middle-aged thymus. **f,** Distribution of up-regulated glycopeptides with different fold change ranges. **g, h,** The numbers of glycopeptides with at least a 5-fold increase in each glycoprotein (**g**) or even each glycosite (**h**) in the middle-aged thymus. **i,** The criteria used to select the normalized glycosylation changes by eliminating the related protein expression changes. Related to Figure 4i. **j,** The numbers and percentages of differential glycopeptides that were only changed at the glycosylation level. Related to Figure 4.

**
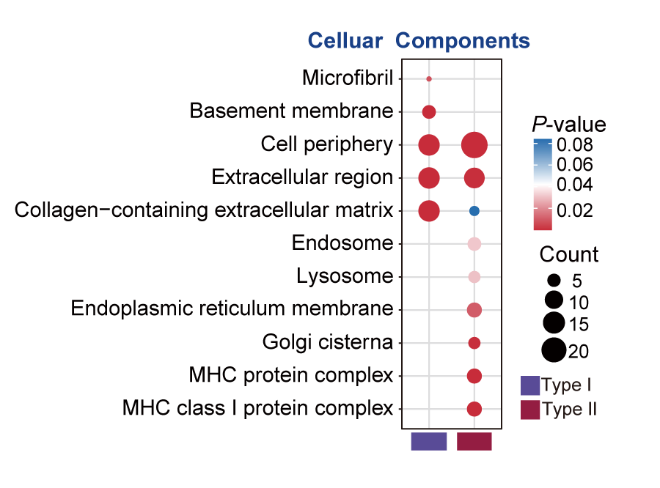
**

**Figure S6. Gene Ontology analysis of proteins carrying increased LacdiNAc glycans in middle-aged mouse thymus.** The bubble diagram shows the cellular components of glycoproteins containing LacdiNAc glycans that were mainly increased at the protein expression level (Type I) or altered at the glycosylation level (Type II). Related to the Figure 5.


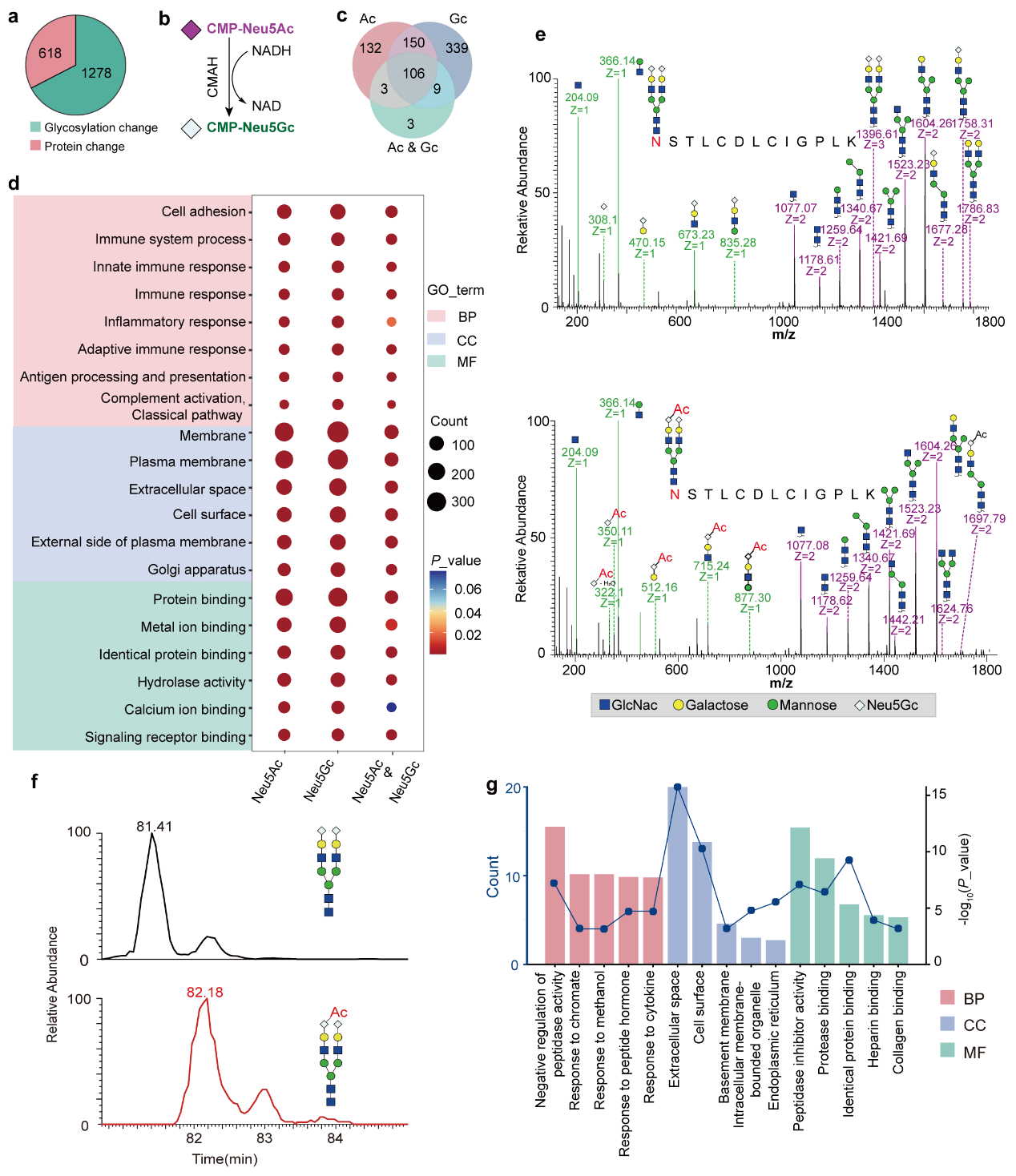


**Figure S7. Sialoglycans and *O*-acetylated sialoglycopeptides (*O*-AcSGPs) were largely up-regulated during mouse thymic involution. a,** Comparison of differential sialylated glycopeptides with or without changes at their corresponding protein-level. **b,** Schematic of the synthesis of Neu5Ac to Neu5Gc. **c,** Venn diagrams showing the comparison of glycoproteins containing Neu5Ac and/or Neu5Gc glycopeptides. **d,** Gene ontology enrichment of glycoproteins containing Neu5Ac or Neu5Gc glycopeptides. **e,** Representative MS/MS spectra of non- (upper panel) and mono- (lower panel) *O*-acetylated glycopeptides identified at Asn-513 of TF. *O*-acetylated sialic acids were identified by their characteristic B/ Y ions (HCD = 27%). **f,** Chromatographic profiles of the glycosite-containing peptide N#STLCDLCIGPLK modified by non- (upper panel) and mono (lower panel) *O*-AcSias. The retention time of the glycopeptides was delayed with each additional *O*-acetyl group. **g,** Gene ontology enrichment analyses of up-regulated *O*-AcSGPs in mouse thymic involution. Related to Figure 6a – 6h.

**
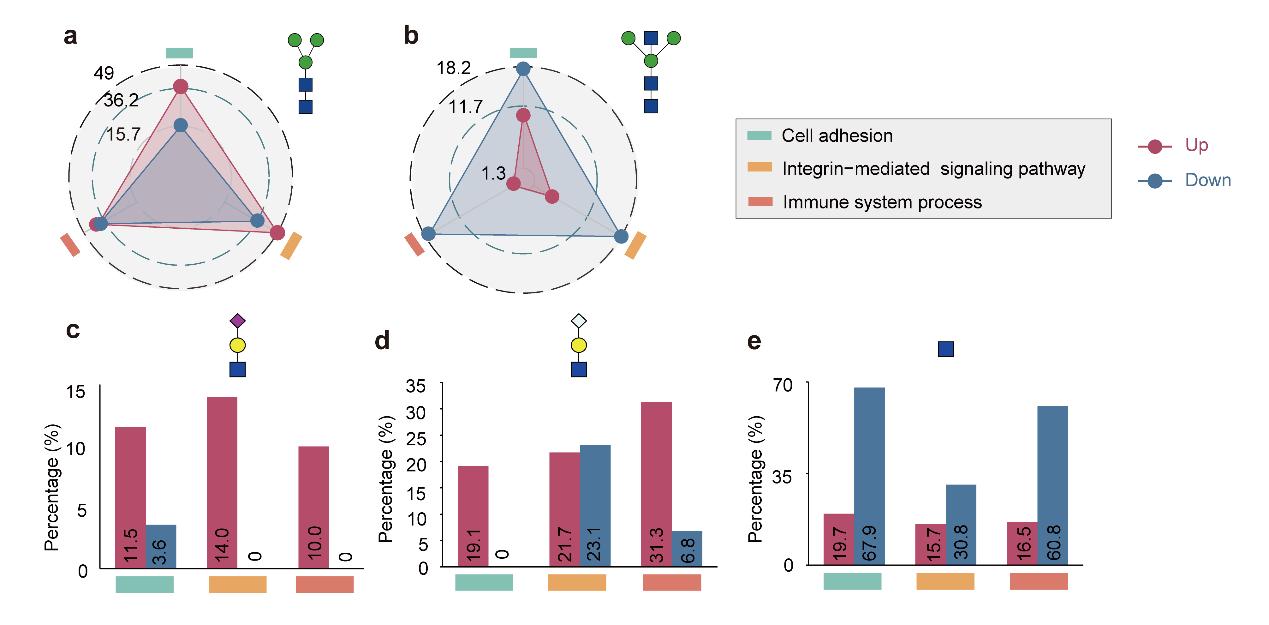
**

**Figure S8. Analyses of glycan sub-structures in biological processes shared by up- and down-regulated glycopeptides. a-e,** Comparison of core-I (**a**), core-III (**b**), sialylated LacNAc(**c**, Neu5Ac; **d**, Neu5Gc), and GlcNAc (**e**) branch structures in biological processes shared by up- and down-regulated glycopeptides. Related to Figure 6i – 6k.

**
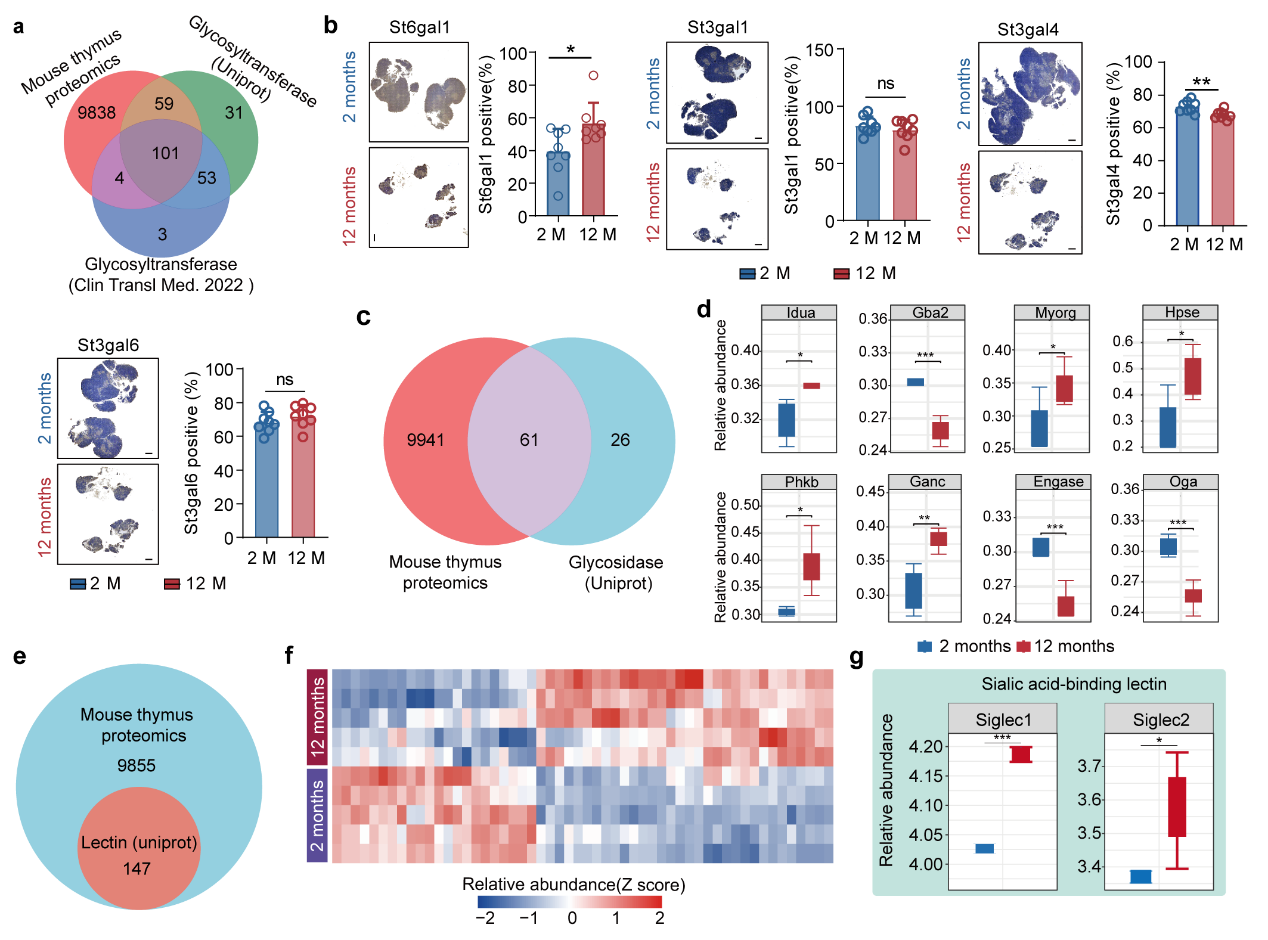
**

**Figure S9. Dysregulation of glycosyltransferases, glycosidases, and glycan-binding proteins during mouse thymic involution. a,** Glycosyltransferases identified in mouse thymus involution proteomics. Glycosyltransferases were based on the Uniprot database and published studies^2^. **b,** Immunohistochemistry on protein expressions of four sialyltransferases in mouse thymus tissues (Scale bar, 500 μm), and the statistical analysis of IHC (n = 8). Data presented as mean ± SD. *P* < 0.05 was considered significant. **c,** Glycosidases identified in mouse thymus involution based on the proteomics data. The known glycosidase database was downloaded from the Uniprot database. **d,** Boxplots showing 8 glycosidases that were significantly changed with thymic involution. **e,** Glycan-binding proteins (GBPs, Lectins) identified in mouse thymus involution based on the proteomics data. The GBP database was downloaded from the Glycomos database. **f,** Heat map showing significantly altered GBPs during mouse thymic involution. **g,** Boxplots showing the dysregulated expressions of Siglec1 and Siglec 2 during thymic involution. Related to Figure 7a - 7d.


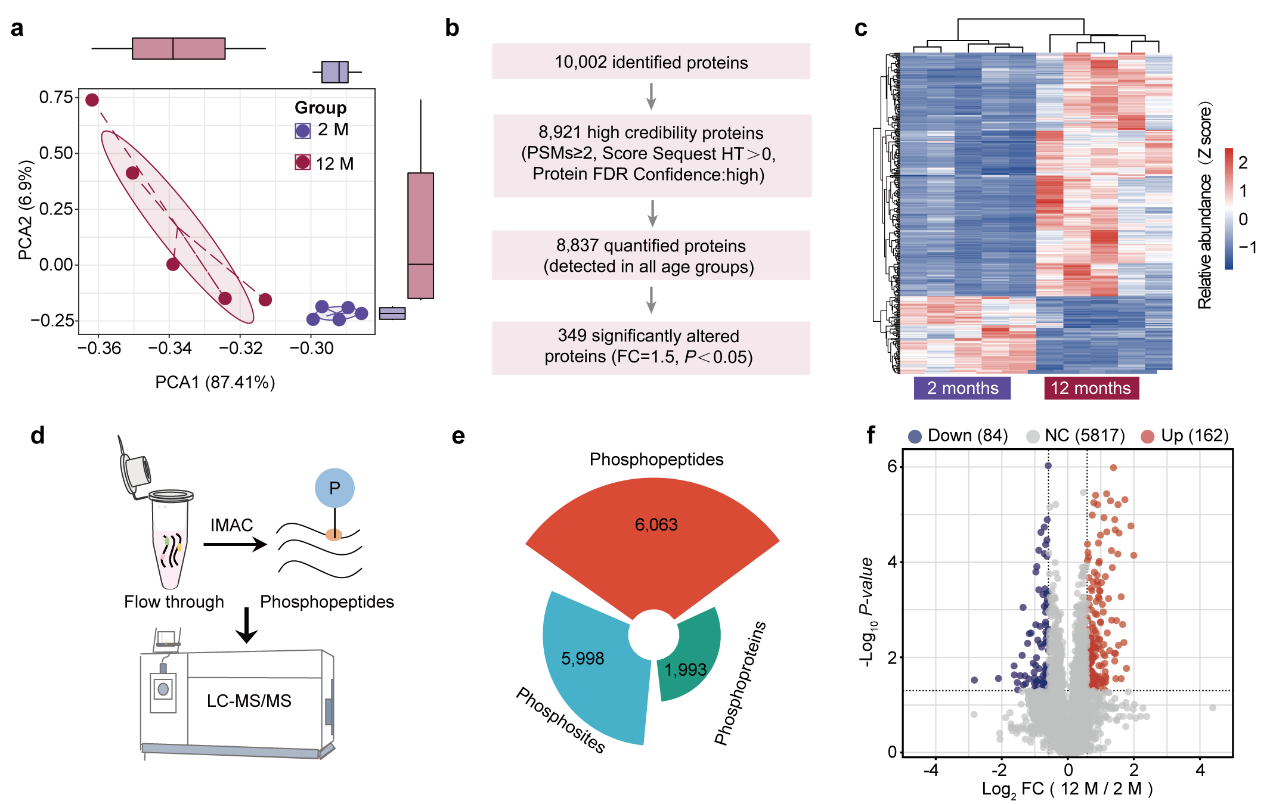


**Figure S10. Proteomic and phosphoproteomic analyses of the mouse thymic involution. a,** Principal component analysis (PCA) of thymus proteome in 2- and 12-month-old mice. **b,** The process for selecting differential proteins associated with mouse thymic involution. **c,** Clustering heatmap showing the differential proteins during thymic involution in mice. **d,** The workflow of phosphoproteomic analysis of mouse thymus. The phosphopeptides were enriched from the flow-through of the MAX columns after intact glycopeptide enrichment. **e,** Total numbers of detected phosphosites, phosphopeptides, and phosphoproteins. **f,** Volcano plot of up- and down-regulated phosphosites during mouse thymic involution. Related to Figure 7e – 7f.
